# Radiomics-based preoperative survival prediction in newly diagnosed glioblastoma: A multicohort study with internal and external validation

**DOI:** 10.1093/noajnl/vdag068

**Published:** 2026-03-13

**Authors:** Toru Umehara, Manabu Kinoshita, Takahiro Sasaki, Junya Fukai, Ema Yoshioka, Daisuke Sakamoto, Kosuke Nakajo, Koji Takano, Hideyuki Arita, Chisato Yokota, Ryuichi Hirayama, Noriyuki Kijima, Yoshiko Okita, Haruhiko Kishima, Yonehiro Kanemura

**Affiliations:** Department of Neurosurgery, Osaka University Graduate School of Medicine, Suita, Osaka, Japan; Department of Neurosurgery, Asahikawa Medical University, Asahikawa, Hokkaido, Japan; Department of Neurological Surgery, School of Medicine, Wakayama Medical University, Wakayama, Wakayama, Japan; Department of Neurological Surgery, School of Medicine, Wakayama Medical University, Wakayama, Wakayama, Japan; Division of Molecular Medicine, Department of Biomedical Research and Innovation, Institute for Clinical Research, NHO Osaka National Hospital, Osaka, Japan; Department of Neurosurgery, Hyogo College of Medicine, Nishinomiya, Hyogo, Japan; Department of Neurosurgery, Osaka Metropolitan University Graduate School of Medicine, Osaka, Japan; Department of Neurosurgery, Kansai Rosai Hospital, Amagasaki, Hyogo, Japan; Department of Neurosurgery, Osaka University Graduate School of Medicine, Suita, Osaka, Japan; Department of Neurosurgery, Osaka International Cancer Institute, Osaka, Japan; Department of Neurosurgery, Osaka University Graduate School of Medicine, Suita, Osaka, Japan; Department of Neurosurgery, Osaka University Graduate School of Medicine, Suita, Osaka, Japan; Department of Neurosurgery, Osaka University Graduate School of Medicine, Suita, Osaka, Japan; Department of Neurosurgery, Osaka University Graduate School of Medicine, Suita, Osaka, Japan; Department of Neurosurgery, Faculty of Medicine, University of Miyazaki, Kiyotake, Miyazaki, Japan; Department of Neurosurgery, Osaka University Graduate School of Medicine, Suita, Osaka, Japan; Division of Molecular Medicine, Department of Biomedical Research and Innovation, Institute for Clinical Research, NHO Osaka National Hospital, Osaka, Japan; Division of Regenerative Medicine, Department of Biomedical Research and Innovation, Institute for Clinical Research, NHO Osaka National Hospital, Osaka, Japan; Department of Neurosurgery, NHO Osaka National Hospital, Osaka, Japan

**Keywords:** glioblastoma, machine learning, radiomics, survival prediction

## Abstract

**Background:**

Preoperative survival prediction in newly diagnosed glioblastoma (nGBM) remains challenging due to limited robustness and standardization across radiomic approaches. We aimed to validate a machine learning–based prognostic model using preoperative MR images and assess its generalizability.

**Methods:**

Two independent cohorts were analyzed: the Kansai Molecular Diagnosis Network for CNS Tumors (KNBTG) and The Cancer Genome Atlas (TCGA). All cases with available preoperative MR images (T1WI, T2WI, and Gd-T1WI) were included. The KNBTG cohort was divided into a training dataset (TD, *n* = 137) and an internal test dataset (ITD, *n* = 141), while the TCGA cohort served as the external test dataset (ETD, *n* = 105). A total of 489 texture features were extracted. Overall survival (OS) was dichotomized at the median, and predictive modeling was performed using least absolute shrinkage and selection operator regularization. The trained model was validated on ITD and ETD.

**Results:**

Radiomic high-risk status was associated with significantly shorter OS in both ITD and ETD (log-rank *P* < .05) and remained independently prognostic in multivariate Cox analysis. Time-dependent area under the receiver operating characteristic curves was consistently higher in models incorporating radiomic-based risk. Of the 13 selected features, “T2_core_GLCMhomogeniety_3_SD” was the only consistent predictor across cohorts and showed strong prognostic stratification, particularly between low- and high-risk groups (cutoff range: 0.0145-0.0180).

**Conclusions:**

Radiomics-based modeling provides reproducible prognostic value in nGBM. The feature “T2_core_GLCMhomogeniety_3_SD” may serve as a reliable imaging biomarker for preoperative risk stratification and individualized treatment planning.

Key Points

- Radiomics-based survival prediction for newly diagnosed glioblastoma is validated across multiple cohorts.
- A single GLCM-based texture feature is an independent, robust prognostic biomarker.
- Combining radiomic risk with age and Karnofsky Performance Status improves preoperative stratification.

Importance of the Study

Radiomics-based survival prediction in newly diagnosed glioblastoma (nGBM) remains challenging because radiomics pipelines vary and external validation is limited. We address this gap by training a machine-learning model using routine preoperative MRI and validating it in internal and external cohorts, demonstrating robust prognostic performance. Beyond model accuracy, we identify a single T2-based GLCM feature that independently stratifies survival risk across cohorts, supporting its role as an imaging biomarker. The model’s texture features likely capture underlying tumor biology and activity present in preoperative images, contributing to accurate prediction. Clinically, integrating the radiomic risk score with age and KPS improves preoperative risk stratification and helps operationalize precision medicine for patients with nGBM.

Glioblastoma, *IDH*-wildtype, remains the most common and aggressive tumor in adult-type diffuse gliomas, with a median overall survival time (MST) of only 15 months.^1^^,^^2^ Given substantial inter-individual variability in prognosis and treatment response, accurate preoperative risk stratification is essential for tailoring the intensity of surgical and adjuvant treatment, thereby minimizing the risks of both undertreatment and overtreatment. Recent advances in molecular biology have provided various insights into the pivotal role of several molecular alterations in risk stratification and therapeutic decision-making of newly diagnosed glioblastoma (nGBM).^3-5^ However, these molecular markers become available, by definition, after tissue sampling; alternatively, the reliable prognostic factors, prior to tissue sampling, are confined to age and KPS score.^6^

Maximal safe resection is generally recommended for nGBM^7^^,^^8^; however, the achievable extent of resection (EOR) and the risk-benefit balance vary across patients depending on tumor location and individual clinical factors. Therefore, additional preoperative determinants beyond age and KPS are needed to support individualized surgical and therapeutic decision-making, partly because advanced age and frailty do not necessarily correlate in contemporary clinical practice. Furthermore, patients’ KPS scores will dramatically alter after surgery, rendering postoperative KPS scores a superior prognostic variable.^9^

By necessity, preoperative imaging in nGBM requires special attention because it is not only useful as a diagnostic stratification modality but may also suggest intrinsic tumor biology and activity. Namely, it has potential clinical significance as a preoperative prognostic factor. For instance, a radiologically localized, superficially located nGBM in a non-eloquent area is more likely to be aggressively resected without neurological deficits, and the patient presenting with such imaging features should have a better prognosis.^10^^,^^11^ In practice, nGBM is thus rarely detected at the earliest stage of development due to its highly proliferative and invasive biology, even though recent advances in neuroradiology have led to an increasing number of incidental brain tumor findings. Most nGBM, at the time of diagnosis, exhibit a wide variety of radiological characteristics represented by tumor necrosis, ring-enhanced tumor area, and peritumoral edema, which often extend into adjacent lobes or the opposite hemisphere. From this, it is difficult to systematically detect their correlation with clinical courses of nGBM based on restricted imaging parameters such as tumor size and location.

Radiomics, that is, high-throughput extraction of quantitative imaging features from routine MRI, has been explored as a means to capture intratumoral heterogeneity and improve prognostic assessment in glioblastoma. Several studies have reported the prognostic value of preoperative MRI-based radiomics for overall survival prediction, including investigations with independent validation and/or evaluation of robustness and generalizability across cohorts.^12-16^ Nevertheless, the reported radiomic signatures and workflows often vary substantially across studies, partly due to differences in feature definitions and preprocessing procedures, which can hinder cross-study comparability and reproducibility. To address these issues, standardization efforts such as the Image Biomarker Standardization Initiative (IBSI) and widely used open-source implementations such as PyRadiomics have been proposed.^17^^,^^18^

In our previous studies, we quantitatively resolved preoperative MR images into multidimensional texture features using an in-house developed system. Then, we subjected them to machine learning for constructing a prediction model. Accordingly, it has been reported that this nGBM risk stratification by machine learning-based texture analysis, “Radiomics,” proved a better prognostic indicator than the preoperative KPS score.^19^ Furthermore, in a subgroup analysis of a multicenter, randomized phase II clinical trial conducted in Japan for patients with nGBM, application of our prediction model successfully stratified patients into high- and low-risk groups.^20^ Nevertheless, our initial studies lacked validation of the prediction model, highlighting the need for further evaluation using independent nGBM cohorts. Given the current emphasis on reproducibility and generalizability in radiomics, independent validation across heterogeneous cohorts is essential for establishing the clinical utility of such models. This study was therefore designed to validate the prognostic utility of our radiomics-based stratification in patients with nGBM.

## Methods

### Eligible Patients

Two cohorts of patients with nGBM were collected for this study. One is a cohort consisting of cases registered to the Kansai Molecular Diagnosis Network for CNS tumors (KNBTG), and the other is the Cancer Genome Atlas (TCGA) cohort; the former is exclusively composed of East Asians, whereas the latter is a mixed-ethnicity cohort. The KNBTG cohort was split into the cases included in our previous work on radiomics for nGBM^19^ and the otherwise newly participating cases to exclude arbitrariness from case selection; the former was regarded as a training dataset, and the latter was an internal test dataset. The nGBM cases from the TCGA cohort were, in turn, defined as an external test dataset.

The initial inclusion criteria were as follows: histopathological diagnosis of newly diagnosed *IDH*-wild-type GBM based on the 2021 WHO Classification of Tumors of the Central Nervous System, preoperative MR images, including T1-weighted images (WI), T2WI, and gadolinium-enhanced (Gd) T1WI, available for radiomics, and uncensored cases or censored cases with the follow-up period of at least 3 months after initial surgery.

### Data Acquisition

The KNBTG, a consortium for malignant brain tumors in the Kansai area of Western Japan, routinely collects glioma samples and clinical information from the affiliated institutions. All tissue specimens were obtained at the time of the initial surgery. Tumor genomic DNA was extracted, and the mutational status of *IDH1/2* and the *TERT* promoter was determined using the Sanger technique. The methylation status of the *MGMT* promoter was assessed using quantitative methylation-specific PCR (qMSP) following the bisulfite modification of tumor genomic DNA. The Sanger technique and qMSP details are described in the online supplementary information (SI) and Tables A1 and A2.^21^ In this study, preoperative MR scans performed using either 1.5- or 3.0-T MRI scanners according to the protocols in each institution were additionally acquired for radiomics. In accordance with the principles of the Helsinki Declaration, approval was obtained from the internal ethical review boards of Osaka University Graduate School of Medicine (approval number: 13244), Osaka National Hospital (No. 713), and all collaborative institutes. Written informed consent was obtained from all patients. Patients still alive at the last follow-up were considered a censored event. The last follow-up was determined with a cutoff date of 30 April 2025. Overall survival (OS) was defined as the time from the date of initial surgery for diagnosis to the date of death or the last follow-up. As for the TCGA-nGBM cohort, the clinical information and *IDH1/2* status were collected from cBioPortal for Cancer Genomics (http://cbioportal.org),^22^ while the MR images (T1WI, T2WI, and Gd-T1WI) were downloaded from The Cancer Imaging Archive (TCIA) repository for nGBM (TCIA; https://www.cancerimagingarchive.net).

### Radiomics and Statistical Data Analysis

Radiomics was conducted in combination with the Matlab (Mathworks, Natick, MA)-based in-house developed software^19^^,^^20^^,^^23^^,^^24^ and the Oxford Centre for Functional MRI of the Brain (FMRIB) Linear Image Registration Tool (FLIRT) provided in the FMRIB Software Library (FSL).^25-27^ Images in Digital Imaging and Communications in Medicine (DICOM) format were converted to the Neuroimaging Informatics Technology Initiative (NIfTI) format prior to feature extraction. All MRI sequences were spatially aligned using FSL-FLIRT (12 degrees of freedom) based on mutual information to ensure voxel-wise correspondence across modalities. Two distinct volumes of interest (VOIs) were initially delineated: one encompassing the Gd tumor region and the other capturing the peritumoral edema, as visualized on T2-weighted images. Lesions were manually delineated in three dimensions by experienced raters (T.U. and T.S.), with review by a senior rater (M.K.). These VOIs were subsequently co-registered to generate “VOI_core_” and “VOI_edema_.” Intensity normalization was performed by re-allocating voxel intensities to 256 gray levels: for T2WI, the full intensity range was rescaled to 256 gray levels, whereas for T1WI and Gd-T1WI, the top 0.1% of intensities were clipped as high-signal noise and the remaining range was rescaled to 256 gray levels. Subsequent radiomic feature extraction included first-order statistics, second-order texture features derived from the gray-level co-occurrence matrix (GLCM) and gray-level run length matrix (GLRLM), and shape characteristics of the VOIs, computed on T1WI, T2WI, Gd-T1WI, T2Edge, and Gdzscore images. For location analysis, images and VOIs were registered to the Montreal Neurological Institute 152 standard brain template (MNI152) to express anatomical location descriptors in a common coordinate system across subjects. The radiomic texture and shape features were implemented based on standard definitions and are reported with full parameter settings; where applicable, feature names are mapped to IBSI nomenclature in Table A3. The overall image analysis workflow is depicted in Figure S1. A comprehensive description of the pipeline for radiomics is also provided in the SI and our previous studies.^19^^,^^20^^,^^23^ A total of 489 texture features, including first-order texture features, second-order features (Gray level co-occurrence matrix and Gray level run length matrix), and shape characteristics of the VOIs, were conclusively collected from each subject. We used Uniform Manifold Approximation and Projection (UMAP) to visualize the high-dimensional radiomic feature space and to qualitatively assess cohort similarity and potential dataset shift. This visualization was exploratory only and was not used for model training or feature selection.

This computational radiomics algorithm, however, has a technical limitation in the complete acquisition of texture features, albeit only in a minority of eligible cases. Specifically, a case harboring multifocal tumors without a connection between enhancing lesions was more likely to be unsuccessful in completely acquiring texture features, while multifocality itself was not a pre-specified exclusion criterion. Then, we used a listwise deletion for statistical analysis; failure cases with one or more missing radiomic features were excluded from subsequent predictive modeling. Datasets after this listwise deletion were termed “D1” from the training dataset, “D2” from the internal test dataset, and “D3” from the external test dataset.

### Development and Validation of the Survival Prediction Model via Radiomics

#### Step 1: Prognostic modeling based on the training dataset

Prognostic modeling via radiomics was attempted using calculations on the glmnet R package. We fit a least absolute shrinkage and selection operator (LASSO)-regularized logistic regression model (family = “binomial”) with the default internal standardization of predictors (standardize = TRUE); coefficients were extracted on the original feature scale as returned by glmnet. To train a model capable of stratifying high- and low-risk nGBM on the D1, the acquired 489 radiomic features and dichotomous OS (short or long OS) were regarded as the explanatory and response variables, respectively. The short or long OS was determined using the MST, with a prognosis equal to or greater than the MST being categorized as long OS. The censored cases with a follow-up period less than the MST were excluded from the calculation to avoid ambiguous dichotomous labeling. After excluding these censored cases from the D1, the dataset consisting of the remaining cases was termed D1’ and was adopted for prognostic modeling. The LASSO regularization was employed to prevent overfitting and the tuning parameter λ, which controls the strength of the L1 penalty, was selected by 10-fold cross-validation (λ_min). To reduce split-dependent instability in LASSO-based feature selection and risk labeling, we repeated the 10-fold cross-validation LASSO procedure 99 times using different fold assignments and derived the final high/low-risk classification by majority voting across iterations.^28^^,^^29^

#### Step 2: Risk stratification on the test datasets

The 99 predictive models constructed in STEP 1 were subsequently applied to the datasets D2 and D3. Each patient in these datasets was stratified into either a high- or low-risk category based on the model’s output, a classification herein referred to as Radiomic-Based Risk (RBR). Ninety-nine RBRs were assigned for each patient in D2 and D3. The final RBR designation for each patient was determined by majority voting across the 99 iterations—that is, the risk category that appeared in more than 50 of the iterations was adopted as the definitive RBR.

#### Step 3: Evaluation of the prediction model performance

The prediction model’s performance was assessed using predictive accuracy, precision, and recall, calculated based on the agreement between the RBR and observed dichotomized OS. For each patient in cohorts D2 and D3, the dichotomous OS status (short vs. long) was determined according to the MST specific to each cohort. Clinical and molecular variables—including age, KPS, EOR, postoperative management, *MGMT* promoter methylation status, and *TERT* promoter mutation—were extracted from the database and used as explanatory variables for survival analysis. For statistical purposes, patients were stratified into binary groups according to the following criteria: age (≥65 vs. <65 years), preoperative KPS (≤70 vs. 80-100), EOR (≤90% vs. 90-100%), and postoperative management (with or without radiotherapy [50–65 Gy] combined with concurrent temozolomide).

The prognostic values of these explanatory variables, including the RBR, were assessed and compared with the Wilcoxon test, Kaplan-Meier plotting, and the Cox proportional hazards model. Backward elimination was applied to identify explanatory variables for multivariate Cox analysis. Time-dependent areas under the receiver operating characteristic curve (AUCs) were calculated to evaluate and compare the predictive performance of each model over time. Parenthetically, *TERT* promoter mutation status and EOR were not considered in the Cox regression model for the TCGA cohort due to a lack of information for all or the majority of patients in this dataset. The difference was considered significant if the *P* value was <.05. All statistical analyses were performed using the R software (version 4.5.0; R Project for Statistical Computing, http://www.r-project.org/). Detailed information on the R environment and packages used for statistical analyses is provided in the SI.

## Results

### Datasets for the Predictive Modeling

**Figure 1.**
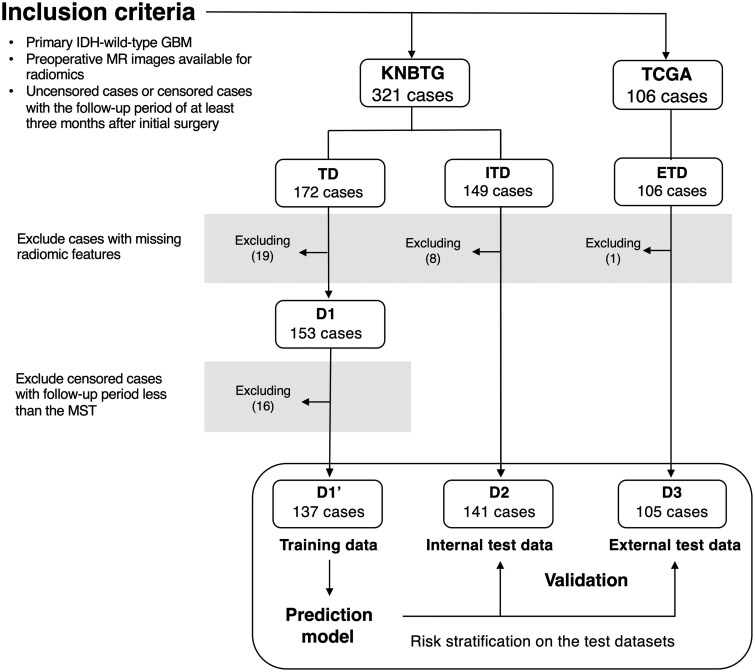
Of the 321 cases initially meeting the inclusion criteria from the KNBTG cohort, 172 were designated as the TD, and the remaining 149 were the ITD. In addition, 106 cases from the TCGA cohort were included as the ETD. After excluding cases with missing radiomic features, 153, 141, and 105 cases were ultimately eligible for prognostic modeling and were assigned to cohorts D1, D2, and D3, respectively. In cohort D1, censored cases with follow-up durations shorter than the MST were excluded, resulting in a final training cohort of 137 cases (D1’). The test cohorts D2 and D3 were used to evaluate the predictive performance of the prognostic model developed using D1’. Abbreviations: ETD, external test dataset; ITD, internal test dataset; KNBTG, Kansai Molecular Diagnosis Network for CNS Tumors; MST, median survival time; TCGA, The Cancer Genome Atlas; TD, training dataset.

**Figure 2.**
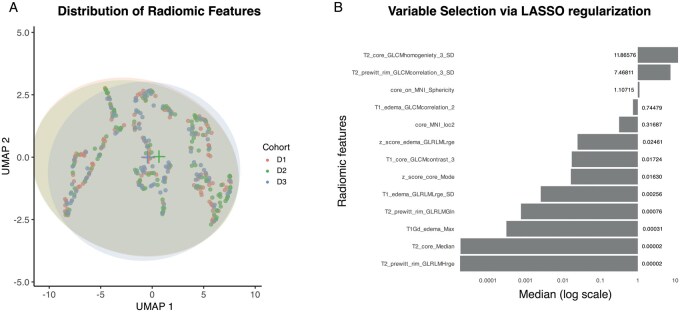
(A) UMAP plot of the radiomic features with confidence ellipses illustrating the distribution of cohorts D1 (red), D2 (green), and D3 (blue). The centers of the ellipses are indicated by plus signs. The near-complete overlap of the ellipses and their centers supports the absence of substantial distributional bias among the cohorts. (B) Radiomic features with a non-zero absolute value of the median coefficient across 99 iterations of model construction were extracted. A total of 13 features were selected, and their absolute median coefficients are visualized as a bar plot on a logarithmic scale.

The study design and case selection process are illustrated in Figure 1. After listwise deletion of cases with missing radiomic data, a total of 153, 141, and 105 cases were included in the prognostic modeling for cohorts D1, D2, and D3, respectively. Detailed clinical, molecular, and radiomic characteristics for each cohort are presented in Tables S1-S3. Comparative analysis using UMAP suggested minimal variation in radiomic feature distributions across the three cohorts, as shown in Figure 2A. Preoperative clinical characteristics and survival outcomes for each dataset are summarized in Table 1. Due to limited data on EOR and *TERT* promoter mutation in D3, these variables were excluded from the Cox regression analysis for that cohort. The MSTs were 16.4, 16.2, and 13.3 months for D1, D2, and D3, respectively. Among the 33 censored cases in D1, 16 had follow-up durations shorter than the MST and were excluded from further analysis. After this exclusion, a total of 137 cases comprised the final training cohort (D1’). For predictive modeling, D1’ was dichotomized into short OS (*n* = 68) and long OS (*n* = 69) groups based on the MST threshold. The test cohorts D2 (*n* = 141) and D3 (*n* = 105) were used to evaluate the model’s predictive performance. Across 99 iterations of model construction, LASSO coefficient profiles and the median coefficients for each radiomic feature are summarized in Table S4. Through this process, 13 radiomic features were selected based on their non-zero median coefficients following LASSO regularization (Figure 2B). Notably, the feature “T2_core_GLCMhomogeniety_3_SD” demonstrated a substantially higher median coefficient, suggesting its prominent contribution to the model. None of the atlas-based location features (i.e., MNI152 occupancy rate of area features) were retained as prognostic factors.

**Table 1.** Comparative clinical characteristics and survival data of each dataset

Datasets		Training dataset		Internal validation dataset	External validation dataset
		D1	D1’	D2	D3
Number of patients		153	137	141	105
Cohort		KNBTG		KNBTG	TCGA
Age at diagnosis (years)	Median	67	68	66	60
	Range	10-93	10-93	14-88	18-85
	Elderly (≥65)	96 (62.7%)	85 (62.0%)	72 (51.1%)	39 (37.1%)
Pre-operative KPS (%)	80–100	85 (55.6%)	77 (56.2%)	72 (51.1%)	69 (80.2%)
	0–70	66 (43.1%)	58 (42.3%)	60 (42.6%)	17 (16.2%)
	N/A	2	2	9	19
EOR	GTR/STR	39 (25.5%)	36 (26.3%)	67(47.5%)	-
	PR/BSY	38 (24.8%)	36 (26.3%)	72(51.1%)	-
	NA	76	65	2	-
TMZ+RT	With	42 (27.5%)	38 (27.7%)	91(64.5%)	32 (30.5%)
	Without	33 (21.6%)	32 (23.3%)	41(29.1%)	59 (56.2%)
	NA	78	67	9	14
MGMT	Met	71 (46.4%)	60 (43.8%)	56(39.7%)	33 (31.4%)
	Unmet	82 (53.6%)	77 (56.2%)	73(51.8%)	39 (37.1%)
	NA	0	0	12	33
TERT promoter	MUT	95 (62.1%)	85 (62.0%)	75(53.2%)	4 (3.8%)
	WT	58 (37.9%)	52 (38.0%)	53(37.6%)	0 (0.0%)
	NA	0	0	13	101
Survival time (months)	Median	16.4	16.0	16.2	13.3
	Range	0.1-73.6	0.1-73.6	0.4-80.6	0.2-51.3

Abbreviations: EOR, extent of resection; KNBTG, Kansai Molecular Diagnosis Network for CNS Tumors; KPS, Karnofsky Performance Status; Met, methylation; TCGA, The Cancer Genome Atlas; TMZ, temozolomide; RT, radiotherapy; Unmet, unmethylation.

### Performance Assessment of the Prediction Model

**Figure 3.**
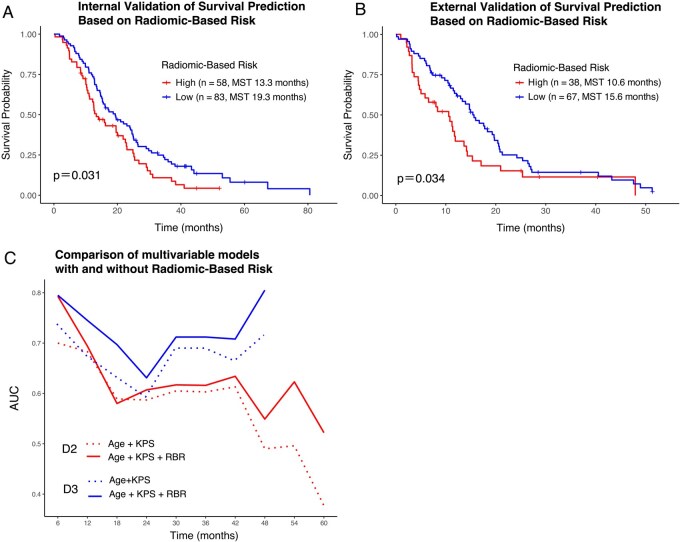
Kaplan-Meier estimates of OS according to the RBR are shown in each cohort: Cohort D2 (*n* = 141) (A) and D3 (*n* = 105) (B). Cases classified as radiomic high-risk significantly showed a worse prognosis than those in the low-risk group in both D2 and D3. Time-dependent AUC analyses at 6-month intervals compare preoperative models with and without RBR (C), incorporating age (≥65 vs. <65 years) and KPS (≤70 vs. 80–100) as predictors. Red and blue lines indicate cohorts D2 and D3, respectively; solid lines denote models with RBR, dashed lines without. Except for a transient point at 18 months in cohort D2, models including RBR consistently demonstrated superior discriminative performance throughout the observation period. Abbreviations: AUC, area under the receiver operating characteristic curve; OS, overall survival; RBR, radiomics-based risk.

The constructed prognostic model based on D1’ was applied to cohorts D2 and D3, categorizing each case as either radiomic high- or low-risk. As shown in the Kaplan-Meier survival curves (Figure 3A and B), patients classified as radiomic high-risk demonstrated significantly shorter survival compared to those in the low-risk group in both D2 (MST: 13.3 vs. 19.3 months; *P* = .031, log-rank test) and D3 (MST: 10.6 vs. 15.6 months; *P* = .034). In univariate Cox regression analysis, radiomic high-risk status was associated with a significantly increased hazard of death, with hazard ratios (HRs) of 1.494 (95% CI: 1.033-2.158; *P* = .035) in D2 and 1.730 (95% CI: 1.109-2.700; *P* = .016) in D3. The prognostic impact of each explanatory variable was further evaluated using Cox regression analysis, as detailed in Table 2. Based on backward stepwise elimination in multivariate modeling, “RBR,” “age category,” “postoperative management,” and “*MGMT* methylation status” were retained in D2, while “RBR” and “age” were selected in D3. In the final multivariate Cox models, the RBR remained an independent predictor of poor survival, with HRs of 1.532 (95% CI: 1.044-2.250; *P* = .029) in D2 and 1.801 (95% CI: 1.153-2.815; *P* = .010) in D3 (Table 2). These findings support the prognostic capability and generalizability of RBR in nGBM.

**Table 2.** Cox proportional hazard models

		Univariate			Multivariate		
		HR	95% CI for HR	*P*	HR	95% CI for HR	*P*
D2 (*n* = 141)							
RBR	High-risk	1.494	1.033-2.158	.035*	1.532	1.044-2.250	.029*
	Low-risk	Ref	-		Ref	-	
Age	≥65	1.511	1.055-2.164	.024*	1.988	1.353-2.921	.001*
	<64	Ref	-		Ref	-	
KPS	≤70%	1.440	0.993-2.089	.055			
	≥80%	Ref	-				
EOR	<90%	1.494	1.041-2.146	.030*	1.631	1.125-2.366	.010*
	≥90%	Ref	-		Ref	-	
TMZ+RT	Without	1.590	1.066-2.373	.023*			
	With	Ref	-				
MGMT	Unmet	1.844	1.245-2.732	.002*	2.125	1.398-3.230	＜.001*
	Met	Ref	-		Ref	-	
TERT promoter	Mutation	1.220	0.827-1.801	.316			
	Wild	Ref	-				
D3 (*n* = 105)							
RBR	High-risk	1.730	1.109-2.700	.016*	1.801	1.153-2.815	.010*
	Low-risk	Ref	-	-	Ref		
Age	≥65	2.108	1.346-3.302	.001*	2.175	1.387-3.409	.001*
	<64	Ref	-	-	Ref		
KPS	≤70%	1.493	0.826-2.698	.184			
	≥80%	Ref	-	-			
TMZ+RT	Without	1.406	0.879-2.251	.155			
	With	Ref	-				
MGMT	Unmet	1.344	0.807-2.240	.256			
	Met	Ref	-				

Abbreviations: CI, confidence interval; HR, hazard ratio; KPS, Karnofsky Performance Status; Met, methylation; TMZ, temozolomide; Ref, Reference, RBR, radiomics-based risk; RT, radiotherapy; Unmet, unmethylation.

* Statistically significant (*P* < .05).

For descriptive purposes, we also evaluated the prediction accuracy for MST-dichotomized OS among cases whose dichotomized outcome could be determined (i.e., excluding cases censored before the MST; 4 cases in D2 and 8 cases in D3). The resulting accuracy was 0.55 in D2 and 0.62 in D3 (Table S5).

Focusing on preoperative prognostic factors, we calculated time-dependent AUCs at 6-month intervals. In cohort D2, the RBR outperformed conventional clinical variables, such as age and KPS, particularly in predicting long-term survival beyond the third year. However, this trend was not evident in cohort D3 (Figure S2). We further compared models with and without the inclusion of RBR. In both D2 and D3, models incorporating RBR consistently yielded superior AUCs across nearly the entire follow-up period (Figure 3C).

### Simplified Prognostic Modeling Using Imaging Features

Using the 13 radiomic features selected through LASSO regularization based on D1’, univariate Cox proportional hazards analyses were performed in cohorts D2 and D3. Among these features, only “T2_core_GLCMhomogeniety_3_SD” was identified as a significant prognostic factor in both cohorts, with lower values associated with poorer survival outcomes (Table S6). The “T2_core_GLCMhomogeniety_3_SD” represents the standard deviation of GLCM homogeniety (IBSI: homogeniety/inverse difference moment) computed within the tumor core VOI on T2-weighted images using an offset distance of 3 voxels across multiple directions. Lower values indicate more directionally consistent (isotropic) texture patterns within the tumor core. This distinctive radiomic feature was designated as the prognostic radiomic feature (PRF).

**Figure 4.**
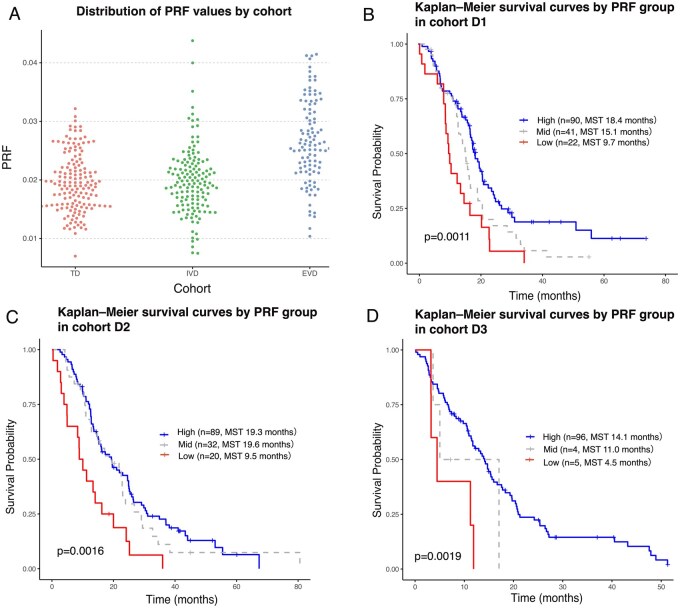
(A) Dot plots illustrate the distribution of PRF values in cohorts D1 (red), D2 (green), and D3 (blue), with slightly higher values observed in cohort D3 compared to D1 and D2. (B-D) Kaplan-Meier survival curves stratified by PRF-based risk groups using the following thresholds: <0.0145, 0.0145 to <0.018, and ≥0.018, are shown for each cohort: D1 (*n* = 153), D2 (*n* = 141), and D3 (*n* = 105), respectively. Log-rank tests revealed statistically significant differences in OS across all cohorts (D1: *P* = .0011; D2: *P* = .0016; D3: *P* = .0192). Abbreviations: PRF, prognostic radiomic feature.

As shown in Figure 4A, the distribution of PRF values varied across the three cohorts, with cohort D3 generally exhibiting higher values. A common cutoff range of 0.0145-0.0180 for PRF was identified across all cohorts, within which the log-rank test yielded statistically significant results (*P* < .05). When stratifying patients into three groups based on PRF values—<0.0145, 0.0145 to < 0.018, and ≥ 0.018—significant differences in survival were observed in each cohort (Figure 4B-D). In pairwise comparisons, the difference between Low and High groups was consistently significant in all cohorts after Bonferroni correction, while Mid groups often showed intermediate but statistically nonsignificant survival patterns. These findings suggest that PRF is a robust prognostic marker, especially when comparing extreme values.

## Discussion

In recent years, the use of machine learning for survival prediction in patients with nGBM has gained increasing attention. In parallel, preoperative MRI–based radiomics has been extensively explored for overall survival prognostication in glioblastoma, and several studies have proposed radiomic signatures and machine-learning models, reporting encouraging risk stratification performance and, in some cases, independent validation and/or robustness assessment.^12-16^ To further improve reproducibility and cross-study comparability, standardization efforts such as the IBSI and widely used open-source implementations such as PyRadiomics have also been advocated.^17^^,^^18^ However, the clinical translation of these models has been hampered by several critical limitations, including insufficient external validation across heterogeneous patient populations, the requirement for large-scale annotated datasets, and the limited interpretability of many predictive algorithms.^30-32^ To address these challenges, this study employed a texture-based radiomic approach designed with an emphasis on model explainability and validated its performance across independent nGBM cohorts.

Our radiomics-based survival prediction model demonstrated strong predictive performance in both the internally validated KNBTG cohort and an externally validated, clinically distinct cohort from TCGA. These findings highlight the reproducibility and generalizability of the RBR model in nGBM and further suggest a potential cohort-independent linkage between specific texture features and the underlying tumor biology. Furthermore, the integration of RBR into preoperative prognostic models consistently improved risk-stratification accuracy. This supports the potential utility of RBR not only as a predictive marker but also as a stratification factor or covariate in future clinical trials targeting nGBM. Such applications may enhance trial design and enable more personalized therapeutic strategies.^33^ This study also identified a single texture feature, “T2_core_GLCMhomogeniety_3_SD,” as a robust and reproducible prognostic biomarker. Lower values of this feature, which indicate more uniform texture within the tumor core on T2-weighted MRI, were consistently linked to poorer outcomes. This observation aligns with the findings reported by Liu et al,^34^ who demonstrated that nGBM exhibiting more uniform and hyperintense regions on Gd-T1WI tended to correlate with worse prognosis. Our study extends this evidence by confirming a similar association on T2WI and validating its prognostic relevance across both internal and external patient cohorts. Importantly, the identification of this single, interpretable radiomic feature offers the potential for developing simplified predictive models that do not rely on complex machine learning algorithms. Future investigations integrating imaging-derived features with genomic and molecular data may further elucidate the biological mechanisms underlying this texture pattern’s strong prognostic significance.

To facilitate the clinical implementation of radiomics-based prognostic models for nGBM, it is essential to ensure transparency in data preprocessing and model development workflows.^31^ In this study, we employed conventional MRI sequences—T1WI, T2WI, and Gd-T1WI—all of which are routinely acquired in standard clinical practice for the evaluation of nGBM. Importantly, our radiomics pipeline does not rely on advanced or nonstandard imaging protocols, thereby enhancing its clinical feasibility. To enable robust quantitative analysis of inherently qualitative image data, we applied a straightforward yet clinically applicable intensity normalization method involving thresholding and window narrowing. Moreover, spatial location features were incorporated into the model, offering additional context that may reflect biologically relevant tumor behavior and regional aggressiveness. Although our previous work employed a supervised principal component analysis framework, its application to survival analysis was limited by the exclusion of censored observations.^19^^,^^20^ To overcome this limitation, we implemented the LASSO regression approach, which effectively accommodates censored data and facilitates interpretable feature selection.^35^^,^^36^ Whereas many previous studies have relied exclusively on internal validation, relatively few have demonstrated external validity to the extent achieved in the present study.^37^^,^^38^ The consistent predictive performance observed across all validation cohorts in our study suggests that the model we developed possesses robust generalizability, capable of adapting to variations in imaging protocols and patient demographics across institutions. Moreover, our approach distinguishes itself from prior models by not relying solely on high-dimensional radiomic features; instead, these features were incorporated into a comprehensive multivariate framework that included well-established postoperative prognostic factors, such as therapeutic context and genomic information. It is particularly noteworthy that radiomics emerged as an independent and statistically significant prognostic factor within this rigorous analytical setting, highlighting its potential clinical utility in optimizing personalized treatment strategies for patients with nGBM.

This study harbors the limitations of a retrospective analysis, and further large-scale prospective studies would be desirable. Moreover, because dichotomizing OS is sensitive to censoring (and requires excluding cases censored before the MST), we primarily interpret our results based on time-to-event analyses (Kaplan-Meier/Cox and time-dependent AUC). Another important limitation lies in the manual delineation of VOIs, which introduces interobserver variability and compromises the reproducibility of texture-derived features. To overcome this limitation, the incorporation of advanced auto-segmentation techniques represents a logical and necessary progression. Recent advances in deep learning–based segmentation models—such as nnU-Net and other convolutional neural network architectures—have shown excellent performance in automatically identifying key regions of nGBM on MRI, including contrast-enhancing areas, necrotic tissue, and surrounding edema.^39^ These techniques not only reduce variability but also improve consistency and scalability, making them highly suitable for large, multi-institutional radiomics studies.

Using machine learning–based texture analysis, we developed a prognostic model for nGBM based on preoperative MR images and validated its performance. The survival analysis showed the effectiveness of the model in both internal and external validation datasets. Notably, we also identified a single GLCM-based texture feature that served as a robust independent prognostic factor. The texture features generated by our algorithm likely reflect underlying biological characteristics and tumor activity embedded within preoperative images of nGBM, thus contributing to accurate predictive modeling. Integrating the RBR with clinical variables such as age and KPS enables more precise preoperative stratification of clinical outcomes in nGBM patients, supporting the advancement of personalized medicine.

## Supplementary Material

vdag068_Supplementary_Data

## Data Availability

The anonymized datasets analyzed in this study are provided in supplementary information.
