# Supplementary material for: Radiomics-based preoperative survival prediction in newly diagnosed glioblastoma: A multicohort study with internal and external validation
Source: Neurooncol Adv. 2026 Mar 13;8(1):vdag068. doi: 10.1093/noajnl/vdag068 (PMC13063838; doi:10.1093/noajnl/vdag068)
Supplement: vdag068_Supplementary_Data [file vdag068_supplementary_data.zip › Supplementary_Data/Supplementary information.docx]

# **Supplementary Information**

# ***The Supplementary Materials are provided as electronic supporting information to enable transparency, reproducibility, and independent validation; their length reflects inclusion of comprehensive raw data tables and detailed feature definitions and is not intended for print.***

# **Title**

# Radiomics-Based Preoperative Survival Prediction in Newly Diagnosed Glioblastoma: A Multicohort Study with Internal and External Validation

**Authors List**

Toru Umehara, Manabu Kinoshita, Takahiro Sasaki, Junya Fukai, Ema Yoshioka, Daisuke Sakamoto, Kosuke Nakajo, Koji Takano, Hideyuki Arita, Chisato Yokota, Ryuichi Hirayama, Noriyuki Kijima, Yoshiko Okita, Haruhiko Kishima, Yonehiro Kanemura

**Corresponding author information**

Corresponding author: Manabu Kinoshita, M.D, Ph.D.

Department of Neurosurgery, Asahikawa Medical University Midorigaoka-higashi 2-1-1-1, Asahikawa, Hokkaido 078-8510, Japan

# Tel: +81-166-68-2594, Fax: +81-166-68-2599, Email: [mail@manabukinoshita.com](mailto:mail@manabukinoshita.com)

# **List of abbreviations**

IBSI, Image Biomarker Standardisation Initiative; KNBTG, Kansai Molecular Diagnosis Network for CNS tumors; LASSO, least absolute shrinkage and selection operator; GBM, glioblastoma; MNI152, the Montreal Neurological Institute's 152 brain template; RBR, Radiomic-Based Risk; time-dependent AUCs; time-dependent area under the receiver operating characteristic curves; UMAP, Uniform Manifold Approximation and Projection; VOI, volume of interest; WI, weighted images

**Supplementary figures**

***Supplementary Figure S1.*** Workflow for VOI co-registration, normalization, and radiomic feature extraction

***Supplementary Figure S2.*** Predictive performance of individual preoperative factors

**Supplementary figure Legends**

***Supplementary Figure S1.*** Illustration showing the workflow for image analysis. Two types of VOIs were created based on Gd enhancement of the tumor and edema lesion identification on T2-weighted images. Both VOIs were co-registered, and VOI core and VOI edema were generated. Subsequently, intensity normalization of all images was performed, and first-order and second-order texture analysis, VOI shape analysis, and location analysis were performed.

***Supplementary Figure S2.*** Time-dependent AUCs were compared at six-month intervals—up to 5 years in cohort D2 (A) and up to 4 years in cohort D3 (B)—using the preoperative prognostic factors: age (≥65 vs. <65 years), KPS (≤70 vs. 80–100), and RBR. In cohort D2, Radiomic-Based Risk showed superior predictive performance for survival, particularly beyond 3 years. In cohort D3, the AUCs for Radiomic-Based Risk consistently outperformed those of KPS across all time points, but remained inferior to age.

**Supplementary tables**

***Supplementary Table S1.*** Training dataset of cohorts D1 (n=153)

***Supplementary Table S2.*** Internal test dataset of cohorts D2 (n=141)

***Supplementary Table S3.*** External test dataset of cohorts D3 (n=105)

***Supplementary Table S4.*** LASSO coefficient profiles and the median coefficients for each radiomic feature

***Supplementary Table S5.*** Performance of radiomic risk classification for dichotomized overall survival in two validation cohorts

***Supplementary Table S6.*** Prognostic impact of selected radiomic features using Univariate Cox proportional hazards analyses

**These tables are provided as a separate Excel file uploaded under the Supplementary Materials section.**

# **Supplementary Methods**

## ***1. Molecular analysis in the KNBTG cohort***

### ***Sanger sequence***

Regions of interest for each gene were amplified from genomic DNA using gene-specific primers (**Supplementary Appendix Table A1**) with AmpliTaq Gold 360 Master Mix (Thermo Fisher Scientific, Waltham, MA) on either a GeneAmp PCR System 9700 or Veriti Thermal Cycler (both from Thermo Fisher Scientific). PCR products were purified using ExoSAP-IT (Thermo Fisher Scientific) and subsequently sequenced with the corresponding sequencing primers (**Supplementary Appendix Table A1**) using the BigDye® Terminator v1.1 Cycle Sequencing Kit (Thermo Fisher Scientific). Sequencing was performed on an ABI 3130xL or 3500 Genetic Analyzer (Thermo Fisher Scientific).

### ***MGMT promoter methylation***

The methylation status of the *MGMT* promoter was assessed using quantitative methylation specific PCR (qMSP). Purified DNA was subjected to bisulfite modification by an EZ DNA Methylation-Gold Kit (Zymo Research, Irvine, CA), according to the manufacturer’s instructions. The qMSP was performed on a QuantStudio12K Flex Real-Time PCR System (Thermo Fisher Scientific) with POWER SYBR® Green PCR Master Mix (Thermo Fisher Scientific). The bisulfite-modified DNA was amplified using specific primer for each methylated or unmethylated molecule as listed in **Supplementary Appendix Table A2**. Real-time PCR conditions were 95 °C for 10 min followed by 45 cycles of 95 °C for 15 s, 60 °C for 60 s. The quantification of methylated and unmethylated sequences was performed by employing the standard curve method as previously described. In dissociation curve analysis, heterogeneity of the amplified methylated and unmethylated molecules was assessed from melting temperature. The mean ± standard deviation of methylation value was calculated from triplicate PCRs. We used 1% cut-off value for the determination of *MGMT* methylation based on an outcome-based study of newly diagnosed GBMs.

## ***Supplementary Appendix Table A1.***

## ***Sequences of primers used for amplification and Sanger sequencing.***

| gene | primer | sequence |
| --- | --- | --- |
| *IDH1* | PCR-forward | 5′- ATATTCTGGGTGGCACGGTC -3′ |
|  | PCR-reverse | 5′- ACAAGTTGGAAATTTCTGGGC -3′ |
| *IDH2* | PCR-forward | 5′- GTTCAAGCTGAAGAAGATGTGG -3′ |
|  | PCR-reverse | 5′- AAGAGGATGGCTAGGCGAGGAG -3′ |
| *TERT* promoter | PCR-forward | 5′- CCTGCCCCTTCACCTTCCAG -3′ |
|  | PCR-reverse | 5′- AGGACGCAGCGCTGCCTGAA -3′ |
| *H3F3A* (for K27 and G34) | PCR-forward | 5′- GGGTAAGTAAGGAGGTCTCTG -3′ |
|  | PCR-reverse | 5′- TACATACAAGAGAGACTTTGTCCC -3′ |
| *HIST1H3B* | PCR-forward | 5′- CTTTCCTTTCCTCCACAGACG -3′ |
|  | PCR-reverse | 5′- TTTGGTAGCGGCGGATCTCG -3′ |

## ***Supplementary Appendix Table A2.***

## ***Sequences of primers used for quantitative methylation specific PCR.***

| gene | primer | sequence |
| --- | --- | --- |
| *MGMT* promoter | M-forward | 5′-TTTCGACGTTCGTAGGTTTTCGC-3′ |
|  | M-reverse | 5′-GCACTCTTCCGAAAACGAAACG-3′ |
|  | U-forward | 5′-TTTGTGTTTTGATGTTTGTAGGTTTTTGT-3′ |
|  | U-reverse | 5′-AACTCCACACTCTTCCAAAAACAAAACA-3′ |
| M, Methylated; U, Unmethylated | | |

## ***2. Radiomics***

Digital Imaging and Communications in Medicine (DICOM) format images were primarily converted to the Neuroimaging Informatics Technology Initiative format (NIfTI) using MRIConvert (University of Oregon Lewis Center for Neuroimaging: http://lcni.uoregon.edu/~jolinda/MRIConvert/), followed by 256 gray-scale level conversion. For T2WI, 100% of the data range was reallocated in 256 gray scale; however, T1WI and Gd-T1WI voxels in the top 0.1% in intensity were deleted as high signal noise, and the remaining 99.9% were reallocated in 256 gray scale, for intensity normalization across all images acquired by different MRI scanners. T2Edge images were constructed by applying a Prewitt filter to T2W images. Gdzscore images were also constructed by performing a voxelwise contrast enhancement calculation using T1WI and Gd-T1WI images. Detailed methods are provided in Supplementary Table S1. A lesion was delineated by manually tracing contrast enhancing lesions on Gd-T1WI and high-intensity lesions on T2WI in three dimensions. These two different voxels of interest (VOIs) were constructed by multiple experienced surgical neuro-oncologists (T.S., T.U., and M.K.); specifically, VOIs creation of the TD by T.S. (already done in the previous study) and of the IVD and EVD by T.U., and review of all the created VOIs by M.K. After VOIs were created, all different image sequences obtained from a single subject were co-registered to each other using a mutual information algorithm with 12 degrees of freedom transformation with FSL-FLIRT to obtain transformation matrices of different image sequences. Three-dimensional lesion VOIs on Gd-T1WI and T2WI were deformed and resliced using the obtained transformation matrices via FSL-FLIRT for each specific image sequence. VOIs created on Gd-T1WI were denoted as “VOIcore,” and the VOIs on T2WI subtracted from VOIs on Gd-T1WI as “VOIedema”. Three different aspects of texture features of the two VOIs were measured on T1WI, T2WI, Gd-T1WI, T2Edge, and Gdzscore image series, i.e., histogram-based first-order texture, second-order texture, and shape characteristics of the VOIs. Furthermore, T2WIs and Gd-T1WIs were registered to a 1.0-mm isotropic, high-resolution T1-weighted brain atlas provided by the Montreal Neurological Institute 152 standard brain template (MNI152) using a mutual information algorithm with 12 degrees of freedom transformation with FSL-FLIRT. VOIcore and VOIedema were then registered onto MNI152 by using the obtained transformation matrices. This procedure was necessary to perform lesion mapping of the VOIs on the standard MNI152 space. It should be noted that tissue classification i.e., delineation of VOIcore and VOIedema was performed prior to spatial normalization and spatial normalization of these VOIs were performed using transformation matrices calculated between the whole brain of the patient and MNI152. Thus, eliminating any contamination of spatial normalization procedure into the process of tissue classification except for the fact that non-linear correction of the distorted brain by the lesion was not performed. The above-mentioned workflow for image analysis is shown in **Supplementary Figure S1**. As listed in **Supplementary Appendix Table A3** below, a total of 489 texture features including first-order texture features, second-order features (Gray level co-occurrence matrix and Grey level run length matrix), and shape characteristics of the VOIs were conclusively collected from each subject.

## ***Statistical Environment and R Packages***

### All statistical analyses were conducted using R software (version 4.5.0; R Foundation for Statistical Computing, Vienna, Austria). The following R packages were employed:

- ggplot2 (version 3.5.2): for data visualization, including Kaplan–Meier survival curves and annotation.
- uwot (version 0.2.3): for performing Uniform Manifold Approximation and Projection (UMAP) to reduce the dimensionality of radiomic features and visualize cohort-level variation.
- ggforce (version 0.5.0): for enhanced plotting capabilities in UMAP visualizations.
- glmnet (version 4.1.8): for implementing a LASSO-regularized logistic regression model (family = "binomial") to select prognostic radiomic features.
- timeROC (version 0.4): for computing time-dependent area under the receiver operating characteristic curve (AUC) at 1, 2, and 3 years to assess model performance.
- survival (version 3.8.3): for Kaplan–Meier estimation, Cox regression modeling, and stepwise log-rank testing to determine optimal cutoff values.
- dplyr (versions 1.1.4) and readr (versions 2.1.5): for data manipulation and import, respectively.

## ***Cutoff Selection for Radiomic Features***

To determine optimal cutoff values for continuous radiomic features, a stepwise log-rank test analysis was performed using the survival package. Candidate cutoff values were generated at 0.0005 intervals. A cutoff was considered valid if it yielded statistically significant differences in overall survival (p < 0.05) across all cohorts (D1–D3), and if group sizes remained balanced (i.e., at least 5 cases per group)

**Supplementary Appendix Table A3. Mapping between radiomic feature names and IBSI nomenclature**

*Note: In the radiomic designation code, the feature term follows IBSI terminology where applicable; prefixes indicate MRI sequence and VOI (core/edema), and suffixes denote parameterization (e.g., distance and aggregation). Features without a direct IBSI equivalent are defined in the “Contents of values” column.*

|  | Radiomicdesignation code | Contents of values |
| --- | --- | --- |
| 1 | T1Gd_original_Total_Surface_Area | Total surface area (*A*) of T1Gd area. |
| 2 | T1Gd_original_Total_Volume | Total volume (*V*) of T1Gd area. |
| 3 | T1Gd_original_Compactness01 | Value calculated by the following equation of T1Gd area; |
| 4 | T1Gd_original_Compactness02 | Value calculated by the following equation of T1Gd area; |
| 5 | T1Gd_original_Spherical_Disporoportion | Value calculated by the following equation of T1Gd area; |
| 6 | T1Gd_original_Sphericity | Value calculated by the following equation of T1Gd area; |
| 7 | T1Gd_original_Surface_to_Volume_ratio | Value calculated by the following equation of T1Gd area; |
| 8 | T2_original_Total_Surface_Area | Total surface area (*A*) of T2 area. |
| 9 | T2_original_Total_Volume | Total volume (*V*) of T2 area. |
| 10 | T2_original_Compactness01 | Value calculated by the following equation of T2 area; |
| 11 | T2_original_Compactness02 | Value calculated by the following equation of T2 area; |
| 12 | T2_original_Spherical_Disporoportion | Value calculated by the following equation of T2 area; |
| 13 | T2_original_Sphericity | Value calculated by the following equation of T2 area; |
| 14 | T2_original_Surface_to_Volume_ratio | Value calculated by the following equation of T2 area; |
| 15 | core_MNI_loc0 | Occupancy rate of area “0” of the MNI structural atlas within the core VOI. This area represents white matter. |
| 16 | core_MNI_loc1 | Occupancy rate of area “1” of the MNI structural atlas within the core VOI. This area represents lateral ventricles. |
| 17 | core_MNI_loc2 | Occupancy rate of area “2” of the MNI structural atlas within the core VOI. This area represents the cerebrum. |
| 18 | core_MNI_loc3 | Occupancy rate of area “3” of the MNI structural atlas within the core VOI. This area represents the frontal lobe. |
| 19 | core_MNI_loc4 | Occupancy rate of area “4” of the MNI structural atlas within the core VOI. This area represents the insular lobe. |
| 20 | core_MNI_loc5 | Occupancy rate of area “5” of the MNI structural atlas within the core VOI. This area represents the occipital lobe. |
| 21 | core_MNI_loc6 | Occupancy rate of area “6” of the MNI structural atlas within the core VOI. This area represents the parietal lobe. |
| 22 | core_MNI_loc7 | Occupancy rate of area “7” of the MNI structural atlas within the core VOI. This area represents the basal ganglia. |
| 23 | core_MNI_loc8 | Occupancy rate of area “8” of the MNI structural atlas within the core VOI. This area represents the temporal lobe. |
| 24 | core_MNI_loc9 | Occupancy rate of area “9” of the MNI structural atlas within the core VOI. This area represents the thalamus. |
| 25 | edema_MNI_loc0 | Occupancy rate of area “0” of the MNI structural atlas within the edema VOI. This area represents white matter. |
| 26 | edema_MNI_loc1 | Occupancy rate of area “1” of the MNI structural atlas within the edema VOI. This area represents lateral ventricles. |
| 27 | edema_MNI_loc2 | Occupancy rate of area “2” of the MNI structural atlas within the edema VOI. This area represents the cerebrum. |
| 28 | edema_MNI_loc3 | Occupancy rate of area “3” of the MNI structural atlas within the edema VOI. This area represents the frontal lobe. |
| 29 | edema_MNI_loc4 | Occupancy rate of area “4” of the MNI structural atlas within the edema VOI. This area represents the insular lobe. |
| 30 | edema_MNI_loc5 | Occupancy rate of area “5” of the MNI structural atlas within the edema VOI. This area represents the occipital lobe. |
| 31 | edema_MNI_loc6 | Occupancy rate of area “6” of the MNI structural atlas within the edema VOI. This area represents the parietal lobe. |
| 32 | edema_MNI_loc7 | Occupancy rate of area “7” of the MNI structural atlas within the edema VOI. This area represents the basal ganglia. |
| 33 | edema_MNI_loc8 | Occupancy rate of area “8” of the MNI structural atlas within the edema VOI. This area represents the temporal lobe. |
| 34 | edema_MNI_loc9 | Occupancy rate of area “9” of the MNI structural atlas within the edema VOI. This area represents the thalamus. |
| 35 | T1Gd_core_Mean | Mean of VOI_core in 256-leveled Gadolinium enhanced T1-weighted image. |
| 36 | T1Gd_core_SD | Standard deviation of VOI_core in 256-leveled Gadolinium enhanced T1-weighted image. |
| 37 | T1Gd_core_Var | Variance of VOI_core in 256-leveled Gadolinium enhanced T1-weighted image. |
| 38 | T1Gd_core_RMS | Root Mean Square of VOI_core in 256-leveled Gadolinium enhanced T1-weighted image. |
| 39 | T1Gd_core_Max | Maximum of VOI_core in 256-leveled Gadolinium enhanced T1-weighted image. |
| 40 | T1Gd_core_Min | Minimum of VOI_core in 256-leveled Gadolinium enhanced T1-weighted image. |
| 41 | T1Gd_core_Median | Median of VOI_core in 256-leveled Gadolinium enhanced T1-weighted image. |
| 42 | T1Gd_core_Mode | Mode of VOI_core in 256-leveled Gadolinium enhanced T1-weighted image. |
| 43 | T1Gd_core_Entropy | Entropyof VOI_core in 256-leveled Gadolinium enhanced T1-weighted image. |
| 44 | T1Gd_core_Kurtosis | Kurtosis of VOI_core in 256-leveled Gadolinium enhanced T1-weighted image. |
| 45 | T1Gd_core_Skewness | Skewness of VOI_core in 256-leveled Gadolinium enhanced T1-weighted image. |
| 46 | T1Gd_core_GLCMcontrast_1 | Mean contrast of GLCM in VOI_core in 256-leveled Gadolinium enhanced T1-weighted image with offset set to 1. |
| 47 | T1Gd_core_GLCMcontrast_2 | Mean contrast of GLCM in VOI_core in 256-leveled Gadolinium enhanced T1-weighted image with offset set to 2. |
| 48 | T1Gd_core_GLCMcontrast_3 | Mean contrast of GLCM in VOI_core in 256-leveled Gadolinium enhanced T1-weighted image with offset set to 3. |
| 49 | T1Gd_core_GLCMenergy_1 | Mean energy of GLCM in VOI_core in 256-leveled Gadolinium enhanced T1-weighted image with offset set to 1. |
| 50 | T1Gd_core_GLCMenergy_2 | Mean energy of GLCM in VOI_core in 256-leveled Gadolinium enhanced T1-weighted image with offset set to 2. |
| 51 | T1Gd_core_GLCMenergy_3 | Mean energy of GLCM in VOI_core in 256-leveled Gadolinium enhanced T1-weighted image with offset set to 3. |
| 52 | T1Gd_core_GLCMhomogeniety_1 | Mean homogeneity of GLCM in VOI_core in 256-leveled Gadolinium enhanced T1-weighted image with offset set to 1. |
| 53 | T1Gd_core_GLCMhomogeniety_2 | Mean homogeneity of GLCM in VOI_core in 256-leveled Gadolinium enhanced T1-weighted image with offset set to 2. |
| 54 | T1Gd_core_GLCMhomogeniety_3 | Mean homogeneity of GLCM in VOI_core in 256-leveled Gadolinium enhanced T1-weighted image with offset set to 3. |
| 55 | T1Gd_core_GLRLMSre | Short run emphasis of GLRLM in VOI_core in 256-leveled Gadolinium enhanced T1-weighted image. |
| 56 | T1Gd_core_GLRLMLre | Long run emphasis of GLRLM in VOI_core in 256-leveled Gadolinium enhanced T1-weighted image. |
| 57 | T1Gd_core_GLRLMGln | GRAY LEVEL NON-UNIFORMITY of GLRLM in VOI_core in 256-leveled Gadolinium enhanced T1-weighted image. |
| 58 | T1Gd_core_GLRLMRp | RUN PERCENTAGE of GLRLM in VOI_core in 256-leveled Gadolinium enhanced T1-weighted image. |
| 59 | T1Gd_core_GLRLMRln | RUN LENGTH NON-UNIFORMITY in VOI_core in 256-leveled Gadolinium enhanced T1-weighted image. |
| 60 | T1Gd_core_GLRLMLrge | LOW GRAY LEVEL RUN EMPHASIS in VOI_core in 256-leveled Gadolinium enhanced T1-weighted image. |
| 61 | T1Gd_core_GLRLMHrge | HIGH GRAY LEVEL RUN EMPHASIS in VOI_core in 256-leveled Gadolinium enhanced T1-weighted image. |
| 62 | T1Gd_core_GLCMcontrast_1_SD | Standard deviation of contrast of GLCM in VOI_core in 256-leveled Gadolinium enhanced T1-weighted image with offset set to 1. |
| 63 | T1Gd_core_GLCMcontrast_2_SD | Standard deviation of contrast of GLCM in VOI_core in 256-leveled Gadolinium enhanced T1-weighted image with offset set to 2. |
| 64 | T1Gd_core_GLCMcontrast_3_SD | Standard deviation of contrast of GLCM in VOI_core in 256-leveled Gadolinium enhanced T1-weighted image with offset set to 3. |
| 65 | T1Gd_core_GLCMenergy_1_SD | Standard deviation of energy of GLCM in VOI_core in 256-leveled Gadolinium enhanced T1-weighted image with offset set to 1. |
| 66 | T1Gd_core_GLCMenergy_2_SD | Standard deviation of energy of GLCM in VOI_core in 256-leveled Gadolinium enhanced T1-weighted image with offset set to 2. |
| 67 | T1Gd_core_GLCMenergy_3_SD | Standard deviation of energy of GLCM in VOI_core in 256-leveled Gadolinium enhanced T1-weighted image with offset set to 3. |
| 68 | T1Gd_core_GLCMhomogeniety_1_SD | Standard deviation of homogeneity of GLCM in VOI_core in 256-leveled Gadolinium enhanced T1-weighted image with offset set to 1. |
| 69 | T1Gd_core_GLCMhomogeniety_2_SD | Standard deviation of homogeneity of GLCM in VOI_core in 256-leveled Gadolinium enhanced T1-weighted image with offset set to 2. |
| 70 | T1Gd_core_GLCMhomogeniety_3_SD | Standard deviation of homogeneity of GLCM in VOI_core in 256-leveled Gadolinium enhanced T1-weighted image with offset set to 3. |
| 71 | T1Gd_core_GLRLMSre_SD | Standard deviation of Short run emphasis of GLRLM in VOI_core in 256-leveled Gadolinium enhanced T1-weighted image. |
| 72 | T1Gd_core_GLRLMLre_SD | Standard deviation of Long run emphasis of GLRLM in VOI_core in 256-leveled Gadolinium enhanced T1-weighted image. |
| 73 | T1Gd_core_GLRLMGln_SD | Standard deviation of GRAY LEVEL NON-UNIFORMITY of GLRLM in VOI_core in 256-leveled Gadolinium enhanced T1-weighted image. |
| 74 | T1Gd_core_GLRLMRp_SD | Standard deviation of RUN PERCENTAGE of GLRLM in VOI_core in 256-leveled Gadolinium enhanced T1-weighted image. |
| 75 | T1Gd_core_GLRLMRln_SD | Standard deviation of RUN LENGTH NON-UNIFORMITY in VOI_core in 256-leveled Gadolinium enhanced T1-weighted image. |
| 76 | T1Gd_core_GLRLMLrge_SD | Standard deviation of LOW GRAY LEVEL RUN EMPHASIS in VOI_core in 256-leveled Gadolinium enhanced T1-weighted image. |
| 77 | T1Gd_core_GLRLMHrge_SD | Standard deviation of HIGH GRAY LEVEL RUN EMPHASIS in VOI_core in 256-leveled Gadolinium enhanced T1-weighted image. |
| 78 | T1Gd_edema_Mean | Mean of VOI_edema in 256-leveled Gadolinium enhanced T1-weighted image. |
| 79 | T1Gd_edema_SD | Standard deviation of VOI_edema in 256-leveled Gadolinium enhanced T1-weighted image. |
| 80 | T1Gd_edema_Var | Variance of VOI_edema in 256-leveled Gadolinium enhanced T1-weighted image. |
| 81 | T1Gd_edema_RMS | Root Mean Square of VOI_edema in 256-leveled Gadolinium enhanced T1-weighted image. |
| 82 | T1Gd_edema_Max | Maximum of VOI_edema in 256-leveled Gadolinium enhanced T1-weighted image. |
| 83 | T1Gd_edema_Min | Minimum of VOI_edema in 256-leveled Gadolinium enhanced T1-weighted image. |
| 84 | T1Gd_edema_Median | Median of VOI_edema in 256-leveled Gadolinium enhanced T1-weighted image. |
| 85 | T1Gd_edema_Mode | Mode of VOI_edema in 256-leveled Gadolinium enhanced T1-weighted image. |
| 86 | T1Gd_edema_Entropy | Entropyof VOI_edema in 256-leveled Gadolinium enhanced T1-weighted image. |
| 87 | T1Gd_edema_Kurtosis | Kurtosis of VOI_edema in 256-leveled Gadolinium enhanced T1-weighted image. |
| 88 | T1Gd_edema_Skewness | Skewness of VOI_edema in 256-leveled Gadolinium enhanced T1-weighted image. |
| 89 | T1Gd_edema_GLCMcontrast_1 | Mean contrast of GLCM in VOI_edema in 256-leveled Gadolinium enhanced T1-weighted image with offset set to 1. |
| 90 | T1Gd_edema_GLCMcontrast_2 | Mean contrast of GLCM in VOI_edema in 256-leveled Gadolinium enhanced T1-weighted image with offset set to 2. |
| 91 | T1Gd_edema_GLCMcontrast_3 | Mean contrast of GLCM in VOI_edema in 256-leveled Gadolinium enhanced T1-weighted image with offset set to 3. |
| 92 | T1Gd_edema_GLCMenergy_1 | Mean energy of GLCM in VOI_edema in 256-leveled Gadolinium enhanced T1-weighted image with offset set to 1. |
| 93 | T1Gd_edema_GLCMenergy_2 | Mean energy of GLCM in VOI_edema in 256-leveled Gadolinium enhanced T1-weighted image with offset set to 2. |
| 94 | T1Gd_edema_GLCMenergy_3 | Mean energy of GLCM in VOI_edema in 256-leveled Gadolinium enhanced T1-weighted image with offset set to 3. |
| 95 | T1Gd_edema_GLCMhomogeniety_1 | Mean homogeneity of GLCM in VOI_edema in 256-leveled Gadolinium enhanced T1-weighted image with offset set to 1. |
| 96 | T1Gd_edema_GLCMhomogeniety_2 | Mean homogeneity of GLCM in VOI_edema in 256-leveled Gadolinium enhanced T1-weighted image with offset set to 2. |
| 97 | T1Gd_edema_GLCMhomogeniety_3 | Mean homogeneity of GLCM in VOI_edema in 256-leveled Gadolinium enhanced T1-weighted image with offset set to 3. |
| 98 | T1Gd_edema_GLRLMSre | Short run emphasis of GLRLM in VOI_edema in 256-leveled Gadolinium enhanced T1-weighted image. |
| 99 | T1Gd_edema_GLRLMLre | Long run emphasis of GLRLM in VOI_edema in 256-leveled Gadolinium enhanced T1-weighted image. |
| 100 | T1Gd_edema_GLRLMGln | GRAY LEVEL NON-UNIFORMITY of GLRLM in VOI_edema in 256-leveled Gadolinium enhanced T1-weighted image. |
| 101 | T1Gd_edema_GLRLMRp | RUN PERCENTAGE of GLRLM in VOI_edema in 256-leveled Gadolinium enhanced T1-weighted image. |
| 102 | T1Gd_edema_GLRLMRln | RUN LENGTH NON-UNIFORMITY in VOI_edema in 256-leveled Gadolinium enhanced T1-weighted image. |
| 103 | T1Gd_edema_GLRLMLrge | LOW GRAY LEVEL RUN EMPHASIS in VOI_edema in 256-leveled Gadolinium enhanced T1-weighted image. |
| 104 | T1Gd_edema_GLRLMHrge | HIGH GRAY LEVEL RUN EMPHASIS in VOI_edema in 256-leveled Gadolinium enhanced T1-weighted image. |
| 105 | T1Gd_edema_GLCMcontrast_1_SD | Standard deviation of contrast of GLCM in VOI_edema in 256-leveled Gadolinium enhanced T1-weighted image with offset set to 1. |
| 106 | T1Gd_edema_GLCMcontrast_2_SD | Standard deviation of contrast of GLCM in VOI_edema in 256-leveled Gadolinium enhanced T1-weighted image with offset set to 2. |
| 107 | T1Gd_edema_GLCMcontrast_3_SD | Standard deviation of contrast of GLCM in VOI_edema in 256-leveled Gadolinium enhanced T1-weighted image with offset set to 3. |
| 108 | T1Gd_edema_GLCMenergy_1_SD | Standard deviation of energy of GLCM in VOI_edema in 256-leveled Gadolinium enhanced T1-weighted image with offset set to 1. |
| 109 | T1Gd_edema_GLCMenergy_2_SD | Standard deviation of energy of GLCM in VOI_edema in 256-leveled Gadolinium enhanced T1-weighted image with offset set to 2. |
| 110 | T1Gd_edema_GLCMenergy_3_SD | Standard deviation of energy of GLCM in VOI_edema in 256-leveled Gadolinium enhanced T1-weighted image with offset set to 3. |
| 111 | T1Gd_edema_GLCMhomogeniety_1_SD | Standard deviation of homogeneity of GLCM in VOI_edema in 256-leveled Gadolinium enhanced T1-weighted image with offset set to 1. |
| 112 | T1Gd_edema_GLCMhomogeniety_2_SD | Standard deviation of homogeneity of GLCM in VOI_edema in 256-leveled Gadolinium enhanced T1-weighted image with offset set to 2. |
| 113 | T1Gd_edema_GLCMhomogeniety_3_SD | Standard deviation of homogeneity of GLCM in VOI_edema in 256-leveled Gadolinium enhanced T1-weighted image with offset set to 3. |
| 114 | T1Gd_edema_GLRLMSre_SD | Standard deviation of Short run emphasis of GLRLM in VOI_edema in 256-leveled Gadolinium enhanced T1-weighted image. |
| 115 | T1Gd_edema_GLRLMLre_SD | Standard deviation of Long run emphasis of GLRLM in VOI_edema in 256-leveled Gadolinium enhanced T1-weighted image. |
| 116 | T1Gd_edema_GLRLMGln_SD | Standard deviation of GRAY LEVEL NON-UNIFORMITY of GLRLM in VOI_edema in 256-leveled Gadolinium enhanced T1-weighted image. |
| 117 | T1Gd_edema_GLRLMRp_SD | Standard deviation of RUN PERCENTAGE of GLRLM in VOI_edema in 256-leveled Gadolinium enhanced T1-weighted image. |
| 118 | T1Gd_edema_GLRLMRln_SD | Standard deviation of RUN LENGTH NON-UNIFORMITY in VOI_edema in 256-leveled Gadolinium enhanced T1-weighted image. |
| 119 | T1Gd_edema_GLRLMLrge_SD | Standard deviation of LOW GRAY LEVEL RUN EMPHASIS in VOI_edema in 256-leveled Gadolinium enhanced T1-weighted image. |
| 120 | T1Gd_edema_GLRLMHrge_SD | Standard deviation of HIGH GRAY LEVEL RUN EMPHASIS in VOI_edema in 256-leveled Gadolinium enhanced T1-weighted image. |
| 121 | T2_core_Mean | Mean of VOI_core in 256-leveled T2-weighted image. |
| 122 | T2_core_SD | Standard deviation of VOI_core in 256-leveled T2-weighted image. |
| 123 | T2_core_Var | Variance of VOI_core in 256-leveled T2-weighted image. |
| 124 | T2_core_RMS | Root Mean Square of VOI_core in 256-leveled T2-weighted image. |
| 125 | T2_core_Max | Maximum of VOI_core in 256-leveled T2-weighted image. |
| 126 | T2_core_Min | Minimum of VOI_core in 256-leveled T2-weighted image. |
| 127 | T2_core_Median | Median of VOI_core in 256-leveled T2-weighted image. |
| 128 | T2_core_Mode | Mode of VOI_core in 256-leveled T2-weighted image. |
| 129 | T2_core_Entropy | Entropyof VOI_core in 256-leveled T2-weighted image. |
| 130 | T2_core_Kurtosis | Kurtosis of VOI_core in 256-leveled T2-weighted image. |
| 131 | T2_core_Skewness | Skewness of VOI_core in 256-leveled T2-weighted image. |
| 132 | T2_core_GLCMcontrast_1 | Mean contrast of GLCM in VOI_core in 256-leveled T2-weighted image with offset set to 1. |
| 133 | T2_core_GLCMcontrast_2 | Mean contrast of GLCM in VOI_core in 256-leveled T2-weighted image with offset set to 2. |
| 134 | T2_core_GLCMcontrast_3 | Mean contrast of GLCM in VOI_core in 256-leveled T2-weighted image with offset set to 3. |
| 135 | T2_core_GLCMenergy_1 | Mean energy of GLCM in VOI_core in 256-leveled T2-weighted image with offset set to 1. |
| 136 | T2_core_GLCMenergy_2 | Mean energy of GLCM in VOI_core in 256-leveled T2-weighted image with offset set to 2. |
| 137 | T2_core_GLCMenergy_3 | Mean energy of GLCM in VOI_core in 256-leveled T2-weighted image with offset set to 3. |
| 138 | T2_core_GLCMhomogeniety_1 | Mean homogeneity of GLCM in VOI_core in 256-leveled T2-weighted image with offset set to 1. |
| 139 | T2_core_GLCMhomogeniety_2 | Mean homogeneity of GLCM in VOI_core in 256-leveled T2-weighted image with offset set to 2. |
| 140 | T2_core_GLCMhomogeniety_3 | Mean homogeneity of GLCM in VOI_core in 256-leveled T2-weighted image with offset set to 3. |
| 141 | T2_core_GLRLMSre | Short run emphasis of GLRLM in VOI_core in 256-leveled T2-weighted image. |
| 142 | T2_core_GLRLMLre | Long run emphasis of GLRLM in VOI_core in 256-leveled T2-weighted image. |
| 143 | T2_core_GLRLMGln | GRAY LEVEL NON-UNIFORMITY of GLRLM in VOI_core in 256-leveled T2-weighted image. |
| 144 | T2_core_GLRLMRp | RUN PERCENTAGE of GLRLM in VOI_core in 256-leveled T2-weighted image. |
| 145 | T2_core_GLRLMRln | RUN LENGTH NON-UNIFORMITY in VOI_core in 256-leveled T2-weighted image. |
| 146 | T2_core_GLRLMLrge | LOW GRAY LEVEL RUN EMPHASIS in VOI_core in 256-leveled T2-weighted image. |
| 147 | T2_core_GLRLMHrge | HIGH GRAY LEVEL RUN EMPHASIS in VOI_core in 256-leveled T2-weighted image. |
| 148 | T2_core_GLCMcontrast_1_SD | Standard deviation of contrast of GLCM in VOI_core in 256-leveled T2-weighted image with offset set to 1. |
| 149 | T2_core_GLCMcontrast_2_SD | Standard deviation of contrast of GLCM in VOI_core in 256-leveled T2-weighted image with offset set to 2. |
| 150 | T2_core_GLCMcontrast_3_SD | Standard deviation of contrast of GLCM in VOI_core in 256-leveled T2-weighted image with offset set to 3. |
| 151 | T2_core_GLCMenergy_1_SD | Standard deviation of energy of GLCM in VOI_core in 256-leveled T2-weighted image with offset set to 1. |
| 152 | T2_core_GLCMenergy_2_SD | Standard deviation of energy of GLCM in VOI_core in 256-leveled T2-weighted image with offset set to 2. |
| 153 | T2_core_GLCMenergy_3_SD | Standard deviation of energy of GLCM in VOI_core in 256-leveled T2-weighted image with offset set to 3. |
| 154 | T2_core_GLCMhomogeniety_1_SD | Standard deviation of homogeneity of GLCM in VOI_core in 256-leveled T2-weighted image with offset set to 1. |
| 155 | T2_core_GLCMhomogeniety_2_SD | Standard deviation of homogeneity of GLCM in VOI_core in 256-leveled T2-weighted image with offset set to 2. |
| 156 | T2_core_GLCMhomogeniety_3_SD | Standard deviation of homogeneity of GLCM in VOI_core in 256-leveled T2-weighted image with offset set to 3. |
| 157 | T2_core_GLRLMSre_SD | Standard deviation of Short run emphasis of GLRLM in VOI_core in 256-leveled T2-weighted image. |
| 158 | T2_core_GLRLMLre_SD | Standard deviation of Long run emphasis of GLRLM in VOI_core in 256-leveled T2-weighted image. |
| 159 | T2_core_GLRLMGln_SD | Standard deviation of GRAY LEVEL NON-UNIFORMITY of GLRLM in VOI_core in 256-leveled T2-weighted image. |
| 160 | T2_core_GLRLMRp_SD | Standard deviation of RUN PERCENTAGE of GLRLM in VOI_core in 256-leveled T2-weighted image. |
| 161 | T2_core_GLRLMRln_SD | Standard deviation of RUN LENGTH NON-UNIFORMITY in VOI_core in 256-leveled T2-weighted image. |
| 162 | T2_core_GLRLMLrge_SD | Standard deviation of LOW GRAY LEVEL RUN EMPHASIS in VOI_core in 256-leveled T2-weighted image. |
| 163 | T2_core_GLRLMHrge_SD | Standard deviation of HIGH GRAY LEVEL RUN EMPHASIS in VOI_core in 256-leveled T2-weighted image. |
| 164 | T2_edema_Mean | Mean of VOI_edema in 256-leveled T2-weighted image. |
| 165 | T2_edema_SD | Standard deviation of VOI_edema in 256-leveled T2-weighted image. |
| 166 | T2_edema_Var | Variance of VOI_edema in 256-leveled T2-weighted image. |
| 167 | T2_edema_RMS | Root Mean Square of VOI_edema in 256-leveled T2-weighted image. |
| 168 | T2_edema_Max | Maximum of VOI_edema in 256-leveled T2-weighted image. |
| 169 | T2_edema_Min | Minimum of VOI_edema in 256-leveled T2-weighted image. |
| 170 | T2_edema_Median | Median of VOI_edema in 256-leveled T2-weighted image. |
| 171 | T2_edema_Mode | Mode of VOI_edema in 256-leveled T2-weighted image. |
| 172 | T2_edema_Entropy | Entropyof VOI_edema in 256-leveled T2-weighted image. |
| 173 | T2_edema_Kurtosis | Kurtosis of VOI_edema in 256-leveled T2-weighted image. |
| 174 | T2_edema_Skewness | Skewness of VOI_edema in 256-leveled T2-weighted image. |
| 175 | T2_edema_GLCMcontrast_1 | Mean contrast of GLCM in VOI_edema in 256-leveled T2-weighted image with offset set to 1. |
| 176 | T2_edema_GLCMcontrast_2 | Mean contrast of GLCM in VOI_edema in 256-leveled T2-weighted image with offset set to 2. |
| 177 | T2_edema_GLCMcontrast_3 | Mean contrast of GLCM in VOI_edema in 256-leveled T2-weighted image with offset set to 3. |
| 178 | T2_edema_GLCMenergy_1 | Mean energy of GLCM in VOI_edema in 256-leveled T2-weighted image with offset set to 1. |
| 179 | T2_edema_GLCMenergy_2 | Mean energy of GLCM in VOI_edema in 256-leveled T2-weighted image with offset set to 2. |
| 180 | T2_edema_GLCMenergy_3 | Mean energy of GLCM in VOI_edema in 256-leveled T2-weighted image with offset set to 3. |
| 181 | T2_edema_GLCMhomogeniety_1 | Mean homogeneity of GLCM in VOI_edema in 256-leveled T2-weighted image with offset set to 1. |
| 182 | T2_edema_GLCMhomogeniety_2 | Mean homogeneity of GLCM in VOI_edema in 256-leveled T2-weighted image with offset set to 2. |
| 183 | T2_edema_GLCMhomogeniety_3 | Mean homogeneity of GLCM in VOI_edema in 256-leveled T2-weighted image with offset set to 3. |
| 184 | T2_edema_GLRLMSre | Short run emphasis of GLRLM in VOI_edema in 256-leveled T2-weighted image. |
| 185 | T2_edema_GLRLMLre | Long run emphasis of GLRLM in VOI_edema in 256-leveled T2-weighted image. |
| 186 | T2_edema_GLRLMGln | GRAY LEVEL NON-UNIFORMITY of GLRLM in VOI_edema in 256-leveled T2-weighted image. |
| 187 | T2_edema_GLRLMRp | RUN PERCENTAGE of GLRLM in VOI_edema in 256-leveled T2-weighted image. |
| 188 | T2_edema_GLRLMRln | RUN LENGTH NON-UNIFORMITY in VOI_edema in 256-leveled T2-weighted image. |
| 189 | T2_edema_GLRLMLrge | LOW GRAY LEVEL RUN EMPHASIS in VOI_edema in 256-leveled T2-weighted image. |
| 190 | T2_edema_GLRLMHrge | HIGH GRAY LEVEL RUN EMPHASIS in VOI_edema in 256-leveled T2-weighted image. |
| 191 | T2_edema_GLCMcontrast_1_SD | Standard deviation of contrast of GLCM in VOI_edema in 256-leveled T2-weighted image with offset set to 1. |
| 192 | T2_edema_GLCMcontrast_2_SD | Standard deviation of contrast of GLCM in VOI_edema in 256-leveled T2-weighted image with offset set to 2. |
| 193 | T2_edema_GLCMcontrast_3_SD | Standard deviation of contrast of GLCM in VOI_edema in 256-leveled T2-weighted image with offset set to 3. |
| 194 | T2_edema_GLCMenergy_1_SD | Standard deviation of energy of GLCM in VOI_edema in 256-leveled T2-weighted image with offset set to 1. |
| 195 | T2_edema_GLCMenergy_2_SD | Standard deviation of energy of GLCM in VOI_edema in 256-leveled T2-weighted image with offset set to 2. |
| 196 | T2_edema_GLCMenergy_3_SD | Standard deviation of energy of GLCM in VOI_edema in 256-leveled T2-weighted image with offset set to 3. |
| 197 | T2_edema_GLCMhomogeniety_1_SD | Standard deviation of homogeneity of GLCM in VOI_edema in 256-leveled T2-weighted image with offset set to 1. |
| 198 | T2_edema_GLCMhomogeniety_2_SD | Standard deviation of homogeneity of GLCM in VOI_edema in 256-leveled T2-weighted image with offset set to 2. |
| 199 | T2_edema_GLCMhomogeniety_3_SD | Standard deviation of homogeneity of GLCM in VOI_edema in 256-leveled T2-weighted image with offset set to 3. |
| 200 | T2_edema_GLRLMSre_SD | Standard deviation of Short run emphasis of GLRLM in VOI_edema in 256-leveled T2-weighted image. |
| 201 | T2_edema_GLRLMLre_SD | Standard deviation of Long run emphasis of GLRLM in VOI_edema in 256-leveled T2-weighted image. |
| 202 | T2_edema_GLRLMGln_SD | Standard deviation of GRAY LEVEL NON-UNIFORMITY of GLRLM in VOI_edema in 256-leveled T2-weighted image. |
| 203 | T2_edema_GLRLMRp_SD | Standard deviation of RUN PERCENTAGE of GLRLM in VOI_edema in 256-leveled T2-weighted image. |
| 204 | T2_edema_GLRLMRln_SD | Standard deviation of RUN LENGTH NON-UNIFORMITY in VOI_edema in 256-leveled T2-weighted image. |
| 205 | T2_edema_GLRLMLrge_SD | Standard deviation of LOW GRAY LEVEL RUN EMPHASIS in VOI_edema in 256-leveled T2-weighted image. |
| 206 | T2_edema_GLRLMHrge_SD | Standard deviation of HIGH GRAY LEVEL RUN EMPHASIS in VOI_edema in 256-leveled T2-weighted image. |
| 207 | T2_prewitt_rim_Mean | Mean of rim of VOI Prewitt filtered 1) 256-leveled T2-weighted image. |
| 208 | T2_prewitt_rim_SD | Standard deviation of Rim of VOI Prewitt filtered 1) 256-leveled T2-weighted image. |
| 209 | T2_prewitt_rim_Var | Variance of Rim of VOI Prewitt filtered 1) 256-leveled T2-weighted image. |
| 210 | T2_prewitt_rim_RMS | Root Mean Square of Rim of VOI Prewitt filtered 1) 256-leveled T2-weighted image. |
| 211 | T2_prewitt_rim_Max | Maximum of Rim of VOI Prewitt filtered 1) 256-leveled T2-weighted image. |
| 212 | T2_prewitt_rim_Min | Minimum of Rim of VOI Prewitt filtered 1) 256-leveled T2-weighted image. |
| 213 | T2_prewitt_rim_Median | Median of Rim of VOI Prewitt filtered 1) 256-leveled T2-weighted image. |
| 214 | T2_prewitt_rim_Mode | Mode of Rim of VOI Prewitt filtered 1) 256-leveled T2-weighted image. |
| 215 | T2_prewitt_rim_Entropy | Entropyof Rim of VOI Prewitt filtered 1) 256-leveled T2-weighted image. |
| 216 | T2_prewitt_rim_Kurtosis | Kurtosis of Rim of VOI Prewitt filtered 1) 256-leveled T2-weighted image. |
| 217 | T2_prewitt_rim_Skewness | Skewness of Rim of VOI Prewitt filtered 1) 256-leveled T2-weighted image. |
| 218 | T2_prewitt_rim_GLCMcontrast_1 | Mean contrast of GLCM in Rim of VOI Prewitt filtered 1) 256-leveled T2-weighted image with offset set to 1. |
| 219 | T2_prewitt_rim_GLCMcontrast_2 | Mean contrast of GLCM in Rim of VOI Prewitt filtered 1) 256-leveled T2-weighted image with offset set to 2. |
| 220 | T2_prewitt_rim_GLCMcontrast_3 | Mean contrast of GLCM in Rim of VOI Prewitt filtered 1) 256-leveled T2-weighted image with offset set to 3. |
| 221 | T2_prewitt_rim_GLCMenergy_1 | Mean energy of GLCM in Rim of VOI Prewitt filtered 1) 256-leveled T2-weighted image with offset set to 1. |
| 222 | T2_prewitt_rim_GLCMenergy_2 | Mean energy of GLCM in Rim of VOI Prewitt filtered 1) 256-leveled T2-weighted image with offset set to 2. |
| 223 | T2_prewitt_rim_GLCMenergy_3 | Mean energy of GLCM in Rim of VOI Prewitt filtered 1) 256-leveled T2-weighted image with offset set to 3. |
| 224 | T2_prewitt_rim_GLCMhomogeniety_1 | Mean homogeneity of GLCM in Rim of VOI Prewitt filtered 1) 256-leveled T2-weighted image with offset set to 1. |
| 225 | T2_prewitt_rim_GLCMhomogeniety_2 | Mean homogeneity of GLCM in Rim of VOI Prewitt filtered 1) 256-leveled T2-weighted image with offset set to 2. |
| 226 | T2_prewitt_rim_GLCMhomogeniety_3 | Mean homogeneity of GLCM in Rim of VOI Prewitt filtered 1) 256-leveled T2-weighted image with offset set to 3. |
| 227 | T2_prewitt_rim_GLRLMSre | Short run emphasis of GLRLM in Rim of VOI Prewitt filtered 1) 256-leveled T2-weighted image. |
| 228 | T2_prewitt_rim_GLRLMLre | Long run emphasis of GLRLM in Rim of VOI Prewitt filtered 1) 256-leveled T2-weighted image. |
| 229 | T2_prewitt_rim_GLRLMGln | GRAY LEVEL NON-UNIFORMITY of GLRLM in Rim of VOI Prewitt filtered 1) 256-leveled T2-weighted image. |
| 230 | T2_prewitt_rim_GLRLMRp | RUN PERCENTAGE of GLRLM in Rim of VOI Prewitt filtered 1) 256-leveled T2-weighted image. |
| 231 | T2_prewitt_rim_GLRLMRln | RUN LENGTH NON-UNIFORMITY in Rim of VOI Prewitt filtered 1) 256-leveled T2-weighted image. |
| 232 | T2_prewitt_rim_GLRLMLrge | LOW GRAY LEVEL RUN EMPHASIS in Rim of VOI Prewitt filtered 1) 256-leveled T2-weighted image. |
| 233 | T2_prewitt_rim_GLRLMHrge | HIGH GRAY LEVEL RUN EMPHASIS in Rim of VOI Prewitt filtered 1) 256-leveled T2-weighted image. |
| 234 | T2_prewitt_rim_GLCMcontrast_1_SD | Standard deviation of contrast of GLCM in Rim of VOI Prewitt filtered 1) 256-leveled T2-weighted image with offset set to 1. |
| 235 | T2_prewitt_rim_GLCMcontrast_2_SD | Standard deviation of contrast of GLCM in Rim of VOI Prewitt filtered 1) 256-leveled T2-weighted image with offset set to 2. |
| 236 | T2_prewitt_rim_GLCMcontrast_3_SD | Standard deviation of contrast of GLCM in Rim of VOI Prewitt filtered 1) 256-leveled T2-weighted image with offset set to 3. |
| 237 | T2_prewitt_rim_GLCMenergy_1_SD | Standard deviation of energy of GLCM in Rim of VOI Prewitt filtered 1) 256-leveled T2-weighted image with offset set to 1. |
| 238 | T2_prewitt_rim_GLCMenergy_2_SD | Standard deviation of energy of GLCM in Rim of VOI Prewitt filtered 1) 256-leveled T2-weighted image with offset set to 2. |
| 239 | T2_prewitt_rim_GLCMenergy_3_SD | Standard deviation of energy of GLCM in Rim of VOI Prewitt filtered 1) 256-leveled T2-weighted image with offset set to 3. |
| 240 | T2_prewitt_rim_GLCMhomogeniety_1_SD | Standard deviation of homogeneity of GLCM in Rim of VOI Prewitt filtered 1) 256-leveled T2-weighted image with offset set to 1. |
| 241 | T2_prewitt_rim_GLCMhomogeniety_2_SD | Standard deviation of homogeneity of GLCM in Rim of VOI Prewitt filtered 1) 256-leveled T2-weighted image with offset set to 2. |
| 242 | T2_prewitt_rim_GLCMhomogeniety_3_SD | Standard deviation of homogeneity of GLCM in Rim of VOI Prewitt filtered 1) 256-leveled T2-weighted image with offset set to 3. |
| 243 | T2_prewitt_rim_GLRLMSre_SD | Standard deviation of Short run emphasis of GLRLM in Rim of VOI Prewitt filtered 1) 256-leveled T2-weighted image. |
| 244 | T2_prewitt_rim_GLRLMLre_SD | Standard deviation of Long run emphasis of GLRLM in Rim of VOI Prewitt filtered 1) 256-leveled T2-weighted image. |
| 245 | T2_prewitt_rim_GLRLMGln_SD | Standard deviation of GRAY LEVEL NON-UNIFORMITY of GLRLM in Rim of VOI Prewitt filtered 1) 256-leveled T2-weighted image. |
| 246 | T2_prewitt_rim_GLRLMRp_SD | Standard deviation of RUN PERCENTAGE of GLRLM in Rim of VOI Prewitt filtered 1) 256-leveled T2-weighted image. |
| 247 | T2_prewitt_rim_GLRLMRln_SD | Standard deviation of RUN LENGTH NON-UNIFORMITY in Rim of VOI Prewitt filtered 1) 256-leveled T2-weighted image. |
| 248 | T2_prewitt_rim_GLRLMLrge_SD | Standard deviation of LOW GRAY LEVEL RUN EMPHASIS in Rim of VOI Prewitt filtered 1) 256-leveled T2-weighted image. |
| 249 | T2_prewitt_rim_GLRLMHrge_SD | Standard deviation of HIGH GRAY LEVEL RUN EMPHASIS in Rim of VOI Prewitt filtered 1) 256-leveled T2-weighted image. |
| 250 | T2_prewitt_rim_GLCMcorrelation_1 | Correlation of GLCM in Rim of VOI Prewitt filtered 1) 256-leveled T2-weighted image with offset set to 1. |
| 251 | T2_prewitt_rim_GLCMcorrelation_2 | Correlation of GLCM in Rim of VOI Prewitt filtered 1) 256-leveled T2-weighted image with offset set to 2. |
| 252 | T2_prewitt_rim_GLCMcorrelation_3 | Correlation of GLCM in Rim of VOI Prewitt filtered 1) 256-leveled T2-weighted image with offset set to 3. |
| 253 | T2_prewitt_rim_GLCMcorrelation_1_SD | Standard deviation of Correlation of GLCM in Rim of VOI Prewitt filtered 1) 256-leveled T2-weighted image with offset set to 1. |
| 254 | T2_prewitt_rim_GLCMcorrelation_2_SD | Standard deviation of Correlation of GLCM in Rim of VOI Prewitt filtered 1) 256-leveled T2-weighted image with offset set to 2. |
| 255 | T2_prewitt_rim_GLCMcorrelation_3_SD | Standard deviation of Correlation of GLCM in Rim of VOI Prewitt filtered 1) 256-leveled T2-weighted image with offset set to 3. |
| 256 | core_on_MNI_Total_Surface_Area | Total surface area (*A*) of core_on_MNI. |
| 257 | core_on_MNI_Total_Volume | Total volume (*V*) of core_on_MNI. |
| 258 | core_on_MNI_Compactness01 | Value calculated by the following equation of core_on_MNI; |
| 259 | core_on_MNI_Compactness02 | Value calculated by the following equation of core_on_MNI; |
| 260 | core_on_MNI_Spherical_Disporoportion | Value calculated by the following equation of core_on_MNI; |
| 261 | core_on_MNI_Sphericity | Value calculated by the following equation of core_on_MNI; |
| 262 | core_on_MNI_Surface_to_Volume_ratio | Value calculated by the following equation of core_on_MNI; |
| 263 | edema_on_MNI_Total_Surface_Area | Total surface area (*A*) of edema_on_MNI. |
| 264 | edema_on_MNI_Total_Volume | Total volume (*V*) of edema_on_MNI. |
| 265 | edema_on_MNI_Compactness01 | Value calculated by the following equation of edema_on_MNI; |
| 266 | edema_on_MNI_Compactness02 | Value calculated by the following equation of edema_on_MNI; |
| 267 | edema_on_MNI_Spherical_Disporoportion | Value calculated by the following equation of edema_on_MNI; |
| 268 | edema_on_MNI_Sphericity | Value calculated by the following equation of edema_on_MNI; |
| 269 | edema_on_MNI_Surface_to_Volume_ratio | Value calculated by the following equation of edema_on_MNI; |
| 270 | T1Gd_edema_GLCMcorrelation_1 | Correlation of GLCM in VOI_edema in 256-leveled Gadolinium enhanced T1-weighted image with offset set to 1. |
| 271 | T1Gd_edema_GLCMcorrelation_2 | Correlation of GLCM in VOI_edema in 256-leveled Gadolinium enhanced T1-weighted image with offset set to 2. |
| 272 | T1Gd_edema_GLCMcorrelation_3 | Correlation of GLCM in VOI_edema in 256-leveled Gadolinium enhanced T1-weighted image with offset set to 3. |
| 273 | T1Gd_edema_GLCMcorrelation_1_SD | Standard deviation of Correlation of GLCM in VOI_edema in 256-leveled Gadolinium enhanced T1-weighted image with offset set to 1. |
| 274 | T1Gd_edema_GLCMcorrelation_2_SD | Standard deviation of Correlation of GLCM in VOI_edema in 256-leveled Gadolinium enhanced T1-weighted image with offset set to 2. |
| 275 | T1Gd_edema_GLCMcorrelation_3_SD | Standard deviation of Correlation of GLCM in VOI_edema in 256-leveled Gadolinium enhanced T1-weighted image with offset set to 3. |
| 276 | T1_core_Mean | Mean of VOI_core in 256-leveled T1-weighted image. |
| 277 | T1_core_SD | Standard deviation of VOI_core in 256-leveled T1-weighted image. |
| 278 | T1_core_Var | Variance of VOI_core in 256-leveled T1-weighted image. |
| 279 | T1_core_RMS | Root Mean Square of VOI_core in 256-leveled T1-weighted image. |
| 280 | T1_core_Max | Maximum of VOI_core in 256-leveled T1-weighted image. |
| 281 | T1_core_Min | Minimum of VOI_core in 256-leveled T1-weighted image. |
| 282 | T1_core_Median | Median of VOI_core in 256-leveled T1-weighted image. |
| 283 | T1_core_Mode | Mode of VOI_core in 256-leveled T1-weighted image. |
| 284 | T1_core_Entropy | Entropyof VOI_core in 256-leveled T1-weighted image. |
| 285 | T1_core_Kurtosis | Kurtosis of VOI_core in 256-leveled T1-weighted image. |
| 286 | T1_core_Skewness | Skewness of VOI_core in 256-leveled T1-weighted image. |
| 287 | T1_core_GLCMcontrast_1 | Mean contrast of GLCM in VOI_core in 256-leveled T1-weighted image with offset set to 1. |
| 288 | T1_core_GLCMcontrast_2 | Mean contrast of GLCM in VOI_core in 256-leveled T1-weighted image with offset set to 2. |
| 289 | T1_core_GLCMcontrast_3 | Mean contrast of GLCM in VOI_core in 256-leveled T1-weighted image with offset set to 3. |
| 290 | T1_core_GLCMenergy_1 | Mean energy of GLCM in VOI_core in 256-leveled T1-weighted image with offset set to 1. |
| 291 | T1_core_GLCMenergy_2 | Mean energy of GLCM in VOI_core in 256-leveled T1-weighted image with offset set to 2. |
| 292 | T1_core_GLCMenergy_3 | Mean energy of GLCM in VOI_core in 256-leveled T1-weighted image with offset set to 3. |
| 293 | T1_core_GLCMhomogeniety_1 | Mean homogeneity of GLCM in VOI_core in 256-leveled T1-weighted image with offset set to 1. |
| 294 | T1_core_GLCMhomogeniety_2 | Mean homogeneity of GLCM in VOI_core in 256-leveled T1-weighted image with offset set to 2. |
| 295 | T1_core_GLCMhomogeniety_3 | Mean homogeneity of GLCM in VOI_core in 256-leveled T1-weighted image with offset set to 3. |
| 296 | T1_core_GLRLMSre | Short run emphasis of GLRLM in VOI_core in 256-leveled T1-weighted image. |
| 297 | T1_core_GLRLMLre | Long run emphasis of GLRLM in VOI_core in 256-leveled T1-weighted image. |
| 298 | T1_core_GLRLMGln | GRAY LEVEL NON-UNIFORMITY of GLRLM in VOI_core in 256-leveled T1-weighted image. |
| 299 | T1_core_GLRLMRp | RUN PERCENTAGE of GLRLM in VOI_core in 256-leveled T1-weighted image. |
| 300 | T1_core_GLRLMRln | RUN LENGTH NON-UNIFORMITY in VOI_core in 256-leveled T1-weighted image. |
| 301 | T1_core_GLRLMLrge | LOW GRAY LEVEL RUN EMPHASIS in VOI_core in 256-leveled T1-weighted image. |
| 302 | T1_core_GLRLMHrge | HIGH GRAY LEVEL RUN EMPHASIS in VOI_core in 256-leveled T1-weighted image. |
| 303 | T1_core_GLCMcontrast_1_SD | Standard deviation of contrast of GLCM in VOI_core in 256-leveled T1-weighted image with offset set to 1. |
| 304 | T1_core_GLCMcontrast_2_SD | Standard deviation of contrast of GLCM in VOI_core in 256-leveled T1-weighted image with offset set to 2. |
| 305 | T1_core_GLCMcontrast_3_SD | Standard deviation of contrast of GLCM in VOI_core in 256-leveled T1-weighted image with offset set to 3. |
| 306 | T1_core_GLCMenergy_1_SD | Standard deviation of energy of GLCM in VOI_core in 256-leveled T1-weighted image with offset set to 1. |
| 307 | T1_core_GLCMenergy_2_SD | Standard deviation of energy of GLCM in VOI_core in 256-leveled T1-weighted image with offset set to 2. |
| 308 | T1_core_GLCMenergy_3_SD | Standard deviation of energy of GLCM in VOI_core in 256-leveled T1-weighted image with offset set to 3. |
| 309 | T1_core_GLCMhomogeniety_1_SD | Standard deviation of homogeneity of GLCM in VOI_core in 256-leveled T1-weighted image with offset set to 1. |
| 310 | T1_core_GLCMhomogeniety_2_SD | Standard deviation of homogeneity of GLCM in VOI_core in 256-leveled T1-weighted image with offset set to 2. |
| 311 | T1_core_GLCMhomogeniety_3_SD | Standard deviation of homogeneity of GLCM in VOI_core in 256-leveled T1-weighted image with offset set to 3. |
| 312 | T1_core_GLRLMSre_SD | Standard deviation of Short run emphasis of GLRLM in VOI_core in 256-leveled T1-weighted image. |
| 313 | T1_core_GLRLMLre_SD | Standard deviation of Long run emphasis of GLRLM in VOI_core in 256-leveled T1-weighted image. |
| 314 | T1_core_GLRLMGln_SD | Standard deviation of GRAY LEVEL NON-UNIFORMITY of GLRLM in VOI_core in 256-leveled T1-weighted image. |
| 315 | T1_core_GLRLMRp_SD | Standard deviation of RUN PERCENTAGE of GLRLM in VOI_core in 256-leveled T1-weighted image. |
| 316 | T1_core_GLRLMRln_SD | Standard deviation of RUN LENGTH NON-UNIFORMITY in VOI_core in 256-leveled T1-weighted image. |
| 317 | T1_core_GLRLMLrge_SD | Standard deviation of LOW GRAY LEVEL RUN EMPHASIS in VOI_core in 256-leveled T1-weighted image. |
| 318 | T1_core_GLRLMHrge_SD | Standard deviation of HIGH GRAY LEVEL RUN EMPHASIS in VOI_core in 256-leveled T1-weighted image. |
| 319 | T1_edema_Mean | Mean of VOI_edema in 256-leveled T1-weighted image. |
| 320 | T1_edema_SD | Standard deviation of VOI_edema in 256-leveled T1-weighted image. |
| 321 | T1_edema_Var | Variance of VOI_edema in 256-leveled T1-weighted image. |
| 322 | T1_edema_RMS | Root Mean Square of VOI_edema in 256-leveled T1-weighted image. |
| 323 | T1_edema_Max | Maximum of VOI_edema in 256-leveled T1-weighted image. |
| 324 | T1_edema_Min | Minimum of VOI_edema in 256-leveled T1-weighted image. |
| 325 | T1_edema_Median | Median of VOI_edema in 256-leveled T1-weighted image. |
| 326 | T1_edema_Mode | Mode of VOI_edema in 256-leveled T1-weighted image. |
| 327 | T1_edema_Entropy | Entropyof VOI_edema in 256-leveled T1-weighted image. |
| 328 | T1_edema_Kurtosis | Kurtosis of VOI_edema in 256-leveled T1-weighted image. |
| 329 | T1_edema_Skewness | Skewness of VOI_edema in 256-leveled T1-weighted image. |
| 330 | T1_edema_GLCMcontrast_1 | Mean contrast of GLCM in VOI_edema in 256-leveled T1-weighted image with offset set to 1. |
| 331 | T1_edema_GLCMcontrast_2 | Mean contrast of GLCM in VOI_edema in 256-leveled T1-weighted image with offset set to 2. |
| 332 | T1_edema_GLCMcontrast_3 | Mean contrast of GLCM in VOI_edema in 256-leveled T1-weighted image with offset set to 3. |
| 333 | T1_edema_GLCMenergy_1 | Mean energy of GLCM in VOI_edema in 256-leveled T1-weighted image with offset set to 1. |
| 334 | T1_edema_GLCMenergy_2 | Mean energy of GLCM in VOI_edema in 256-leveled T1-weighted image with offset set to 2. |
| 335 | T1_edema_GLCMenergy_3 | Mean energy of GLCM in VOI_edema in 256-leveled T1-weighted image with offset set to 3. |
| 336 | T1_edema_GLCMhomogeniety_1 | Mean homogeneity of GLCM in VOI_edema in 256-leveled T1-weighted image with offset set to 1. |
| 337 | T1_edema_GLCMhomogeniety_2 | Mean homogeneity of GLCM in VOI_edema in 256-leveled T1-weighted image with offset set to 2. |
| 338 | T1_edema_GLCMhomogeniety_3 | Mean homogeneity of GLCM in VOI_edema in 256-leveled T1-weighted image with offset set to 3. |
| 339 | T1_edema_GLRLMSre | Short run emphasis of GLRLM in VOI_edema in 256-leveled T1-weighted image. |
| 340 | T1_edema_GLRLMLre | Long run emphasis of GLRLM in VOI_edema in 256-leveled T1-weighted image. |
| 341 | T1_edema_GLRLMGln | GRAY LEVEL NON-UNIFORMITY of GLRLM in VOI_edema in 256-leveled T1-weighted image. |
| 342 | T1_edema_GLRLMRp | RUN PERCENTAGE of GLRLM in VOI_edema in 256-leveled T1-weighted image. |
| 343 | T1_edema_GLRLMRln | RUN LENGTH NON-UNIFORMITY in VOI_edema in 256-leveled T1-weighted image. |
| 344 | T1_edema_GLRLMLrge | LOW GRAY LEVEL RUN EMPHASIS in VOI_edema in 256-leveled T1-weighted image. |
| 345 | T1_edema_GLRLMHrge | HIGH GRAY LEVEL RUN EMPHASIS in VOI_edema in 256-leveled T1-weighted image. |
| 346 | T1_edema_GLCMcontrast_1_SD | Standard deviation of contrast of GLCM in VOI_edema in 256-leveled T1-weighted image with offset set to 1. |
| 347 | T1_edema_GLCMcontrast_2_SD | Standard deviation of contrast of GLCM in VOI_edema in 256-leveled T1-weighted image with offset set to 2. |
| 348 | T1_edema_GLCMcontrast_3_SD | Standard deviation of contrast of GLCM in VOI_edema in 256-leveled T1-weighted image with offset set to 3. |
| 349 | T1_edema_GLCMenergy_1_SD | Standard deviation of energy of GLCM in VOI_edema in 256-leveled T1-weighted image with offset set to 1. |
| 350 | T1_edema_GLCMenergy_2_SD | Standard deviation of energy of GLCM in VOI_edema in 256-leveled T1-weighted image with offset set to 2. |
| 351 | T1_edema_GLCMenergy_3_SD | Standard deviation of energy of GLCM in VOI_edema in 256-leveled T1-weighted image with offset set to 3. |
| 352 | T1_edema_GLCMhomogeniety_1_SD | Standard deviation of homogeneity of GLCM in VOI_edema in 256-leveled T1-weighted image with offset set to 1. |
| 353 | T1_edema_GLCMhomogeniety_2_SD | Standard deviation of homogeneity of GLCM in VOI_edema in 256-leveled T1-weighted image with offset set to 2. |
| 354 | T1_edema_GLCMhomogeniety_3_SD | Standard deviation of homogeneity of GLCM in VOI_edema in 256-leveled T1-weighted image with offset set to 3. |
| 355 | T1_edema_GLRLMSre_SD | Standard deviation of Short run emphasis of GLRLM in VOI_edema in 256-leveled T1-weighted image. |
| 356 | T1_edema_GLRLMLre_SD | Standard deviation of Long run emphasis of GLRLM in VOI_edema in 256-leveled T1-weighted image. |
| 357 | T1_edema_GLRLMGln_SD | Standard deviation of GRAY LEVEL NON-UNIFORMITY of GLRLM in VOI_edema in 256-leveled T1-weighted image. |
| 358 | T1_edema_GLRLMRp_SD | Standard deviation of RUN PERCENTAGE of GLRLM in VOI_edema in 256-leveled T1-weighted image. |
| 359 | T1_edema_GLRLMRln_SD | Standard deviation of RUN LENGTH NON-UNIFORMITY in VOI_edema in 256-leveled T1-weighted image. |
| 360 | T1_edema_GLRLMLrge_SD | Standard deviation of LOW GRAY LEVEL RUN EMPHASIS in VOI_edema in 256-leveled T1-weighted image. |
| 361 | T1_edema_GLRLMHrge_SD | Standard deviation of HIGH GRAY LEVEL RUN EMPHASIS in VOI_edema in 256-leveled T1-weighted image. |
| 362 | T2_edema_GLCMcorrelation_1 | Correlation of GLCM in VOI_edema in 256-leveled T2-weighted image with offset set to 1. |
| 363 | T2_edema_GLCMcorrelation_2 | Correlation of GLCM in VOI_edema in 256-leveled T2-weighted image with offset set to 2. |
| 364 | T2_edema_GLCMcorrelation_3 | Correlation of GLCM in VOI_edema in 256-leveled T2-weighted image with offset set to 3. |
| 365 | T2_edema_GLCMcorrelation_1_SD | Standard deviation of Correlation of GLCM in VOI_edema in 256-leveled T2-weighted image with offset set to 1. |
| 366 | T2_edema_GLCMcorrelation_2_SD | Standard deviation of Correlation of GLCM in VOI_edema in 256-leveled T2-weighted image with offset set to 2. |
| 367 | T2_edema_GLCMcorrelation_3_SD | Standard deviation of Correlation of GLCM in VOI_edema in 256-leveled T2-weighted image with offset set to 3. |
| 368 | z_score_core_Mean | Mean of VOI_core in Gdzscore 2). |
| 369 | z_score_core_SD | Standard deviation of VOI_core in Gdzscore 2). |
| 370 | z_score_core_Var | Variance of VOI_core in Gdzscore 2). |
| 371 | z_score_core_RMS | Root Mean Square of VOI_core in Gdzscore 2). |
| 372 | z_score_core_Max | Maximum of VOI_core in Gdzscore 2). |
| 373 | z_score_core_Min | Minimum of VOI_core in Gdzscore 2). |
| 374 | z_score_core_Median | Median of VOI_core in Gdzscore 2). |
| 375 | z_score_core_Mode | Mode of VOI_core in Gdzscore 2). |
| 376 | z_score_core_Entropy | Entropyof VOI_core in Gdzscore 2). |
| 377 | z_score_core_Kurtosis | Kurtosis of VOI_core in Gdzscore 2). |
| 378 | z_score_core_Skewness | Skewness of VOI_core in Gdzscore 2). |
| 379 | z_score_core_GLCMcontrast_1 | Mean contrast of GLCM in VOI_core in Gdzscore 2) with offset set to 1. |
| 380 | z_score_core_GLCMcontrast_2 | Mean contrast of GLCM in VOI_core in Gdzscore 2) with offset set to 2. |
| 381 | z_score_core_GLCMcontrast_3 | Mean contrast of GLCM in VOI_core in Gdzscore 2) with offset set to 3. |
| 382 | z_score_core_GLCMenergy_1 | Mean energy of GLCM in VOI_core in Gdzscore 2) with offset set to 1. |
| 383 | z_score_core_GLCMenergy_2 | Mean energy of GLCM in VOI_core in Gdzscore 2) with offset set to 2. |
| 384 | z_score_core_GLCMenergy_3 | Mean energy of GLCM in VOI_core in Gdzscore 2) with offset set to 3. |
| 385 | z_score_core_GLCMhomogeniety_1 | Mean homogeneity of GLCM in VOI_core in Gdzscore 2) with offset set to 1. |
| 386 | z_score_core_GLCMhomogeniety_2 | Mean homogeneity of GLCM in VOI_core in Gdzscore 2) with offset set to 2. |
| 387 | z_score_core_GLCMhomogeniety_3 | Mean homogeneity of GLCM in VOI_core in Gdzscore 2) with offset set to 3. |
| 388 | z_score_core_GLRLMSre | Short run emphasis of GLRLM in VOI_core in Gdzscore 2). |
| 389 | z_score_core_GLRLMLre | Long run emphasis of GLRLM in VOI_core in Gdzscore 2). |
| 390 | z_score_core_GLRLMGln | GRAY LEVEL NON-UNIFORMITY of GLRLM in VOI_core in Gdzscore 2). |
| 391 | z_score_core_GLRLMRp | RUN PERCENTAGE of GLRLM in VOI_core in Gdzscore 2). |
| 392 | z_score_core_GLRLMRln | RUN LENGTH NON-UNIFORMITY in VOI_core in Gdzscore 2). |
| 393 | z_score_core_GLRLMLrge | LOW GRAY LEVEL RUN EMPHASIS in VOI_core in Gdzscore 2). |
| 394 | z_score_core_GLRLMHrge | HIGH GRAY LEVEL RUN EMPHASIS in VOI_core in Gdzscore 2). |
| 395 | z_score_core_GLCMcontrast_1_SD | Standard deviation of contrast of GLCM in VOI_core in Gdzscore 2) with offset set to 1. |
| 396 | z_score_core_GLCMcontrast_2_SD | Standard deviation of contrast of GLCM in VOI_core in Gdzscore 2) with offset set to 2. |
| 397 | z_score_core_GLCMcontrast_3_SD | Standard deviation of contrast of GLCM in VOI_core in Gdzscore 2) with offset set to 3. |
| 398 | z_score_core_GLCMenergy_1_SD | Standard deviation of energy of GLCM in VOI_core in Gdzscore 2) with offset set to 1. |
| 399 | z_score_core_GLCMenergy_2_SD | Standard deviation of energy of GLCM in VOI_core in Gdzscore 2) with offset set to 2. |
| 400 | z_score_core_GLCMenergy_3_SD | Standard deviation of energy of GLCM in VOI_core in Gdzscore 2) with offset set to 3. |
| 401 | z_score_core_GLCMhomogeniety_1_SD | Standard deviation of homogeneity of GLCM in VOI_core in Gdzscore 2) with offset set to 1. |
| 402 | z_score_core_GLCMhomogeniety_2_SD | Standard deviation of homogeneity of GLCM in VOI_core in Gdzscore 2) with offset set to 2. |
| 403 | z_score_core_GLCMhomogeniety_3_SD | Standard deviation of homogeneity of GLCM in VOI_core in Gdzscore 2) with offset set to 3. |
| 404 | z_score_core_GLRLMSre_SD | Standard deviation of Short run emphasis of GLRLM in VOI_core in Gdzscore 2). |
| 405 | z_score_core_GLRLMLre_SD | Standard deviation of Long run emphasis of GLRLM in VOI_core in Gdzscore 2). |
| 406 | z_score_core_GLRLMGln_SD | Standard deviation of GRAY LEVEL NON-UNIFORMITY of GLRLM in VOI_core in Gdzscore 2). |
| 407 | z_score_core_GLRLMRp_SD | Standard deviation of RUN PERCENTAGE of GLRLM in VOI_core in Gdzscore 2). |
| 408 | z_score_core_GLRLMRln_SD | Standard deviation of RUN LENGTH NON-UNIFORMITY in VOI_core in Gdzscore 2). |
| 409 | z_score_core_GLRLMLrge_SD | Standard deviation of LOW GRAY LEVEL RUN EMPHASIS in VOI_core in Gdzscore 2). |
| 410 | z_score_core_GLRLMHrge_SD | Standard deviation of HIGH GRAY LEVEL RUN EMPHASIS in VOI_core in Gdzscore 2). |
| 411 | z_score_edema_Mean | Mean of VOI_edema in Gdzscore 2). |
| 412 | z_score_edema_SD | Standard deviation of VOI_edema in Gdzscore 2). |
| 413 | z_score_edema_Var | Variance of VOI_edema in Gdzscore 2). |
| 414 | z_score_edema_RMS | Root Mean Square of VOI_edema in Gdzscore 2). |
| 415 | z_score_edema_Max | Maximum of VOI_edema in Gdzscore 2). |
| 416 | z_score_edema_Min | Minimum of VOI_edema in Gdzscore 2). |
| 417 | z_score_edema_Median | Median of VOI_edema in Gdzscore 2). |
| 418 | z_score_edema_Mode | Mode of VOI_edema in Gdzscore 2). |
| 419 | z_score_edema_Entropy | Entropyof VOI_edema in Gdzscore 2). |
| 420 | z_score_edema_Kurtosis | Kurtosis of VOI_edema in Gdzscore 2). |
| 421 | z_score_edema_Skewness | Skewness of VOI_edema in Gdzscore 2). |
| 422 | z_score_edema_GLCMcontrast_1 | Mean contrast of GLCM in VOI_edema in Gdzscore 2) with offset set to 1. |
| 423 | z_score_edema_GLCMcontrast_2 | Mean contrast of GLCM in VOI_edema in Gdzscore 2) with offset set to 2. |
| 424 | z_score_edema_GLCMcontrast_3 | Mean contrast of GLCM in VOI_edema in Gdzscore 2) with offset set to 3. |
| 425 | z_score_edema_GLCMenergy_1 | Mean energy of GLCM in VOI_edema in Gdzscore 2) with offset set to 1. |
| 426 | z_score_edema_GLCMenergy_2 | Mean energy of GLCM in VOI_edema in Gdzscore 2) with offset set to 2. |
| 427 | z_score_edema_GLCMenergy_3 | Mean energy of GLCM in VOI_edema in Gdzscore 2) with offset set to 3. |
| 428 | z_score_edema_GLCMhomogeniety_1 | Mean homogeneity of GLCM in VOI_edema in Gdzscore 2) with offset set to 1. |
| 429 | z_score_edema_GLCMhomogeniety_2 | Mean homogeneity of GLCM in VOI_edema in Gdzscore 2) with offset set to 2. |
| 430 | z_score_edema_GLCMhomogeniety_3 | Mean homogeneity of GLCM in VOI_edema in Gdzscore 2) with offset set to 3. |
| 431 | z_score_edema_GLRLMSre | Short run emphasis of GLRLM in VOI_edema in Gdzscore 2). |
| 432 | z_score_edema_GLRLMLre | Long run emphasis of GLRLM in VOI_edema in Gdzscore 2). |
| 433 | z_score_edema_GLRLMGln | GRAY LEVEL NON-UNIFORMITY of GLRLM in VOI_edema in Gdzscore 2). |
| 434 | z_score_edema_GLRLMRp | RUN PERCENTAGE of GLRLM in VOI_edema in Gdzscore 2). |
| 435 | z_score_edema_GLRLMRln | RUN LENGTH NON-UNIFORMITY in VOI_edema in Gdzscore 2). |
| 436 | z_score_edema_GLRLMLrge | LOW GRAY LEVEL RUN EMPHASIS in VOI_edema in Gdzscore 2). |
| 437 | z_score_edema_GLRLMHrge | HIGH GRAY LEVEL RUN EMPHASIS in VOI_edema in Gdzscore 2). |
| 438 | z_score_edema_GLCMcontrast_1_SD | Standard deviation of contrast of GLCM in VOI_edema in Gdzscore 2) with offset set to 1. |
| 439 | z_score_edema_GLCMcontrast_2_SD | Standard deviation of contrast of GLCM in VOI_edema in Gdzscore 2) with offset set to 2. |
| 440 | z_score_edema_GLCMcontrast_3_SD | Standard deviation of contrast of GLCM in VOI_edema in Gdzscore 2) with offset set to 3. |
| 441 | z_score_edema_GLCMenergy_1_SD | Standard deviation of energy of GLCM in VOI_edema in Gdzscore 2) with offset set to 1. |
| 442 | z_score_edema_GLCMenergy_2_SD | Standard deviation of energy of GLCM in VOI_edema in Gdzscore 2) with offset set to 2. |
| 443 | z_score_edema_GLCMenergy_3_SD | Standard deviation of energy of GLCM in VOI_edema in Gdzscore 2) with offset set to 3. |
| 444 | z_score_edema_GLCMhomogeniety_1_SD | Standard deviation of homogeneity of GLCM in VOI_edema in Gdzscore 2) with offset set to 1. |
| 445 | z_score_edema_GLCMhomogeniety_2_SD | Standard deviation of homogeneity of GLCM in VOI_edema in Gdzscore 2) with offset set to 2. |
| 446 | z_score_edema_GLCMhomogeniety_3_SD | Standard deviation of homogeneity of GLCM in VOI_edema in Gdzscore 2) with offset set to 3. |
| 447 | z_score_edema_GLRLMSre_SD | Standard deviation of Short run emphasis of GLRLM in VOI_edema in Gdzscore 2). |
| 448 | z_score_edema_GLRLMLre_SD | Standard deviation of Long run emphasis of GLRLM in VOI_edema in Gdzscore 2). |
| 449 | z_score_edema_GLRLMGln_SD | Standard deviation of GRAY LEVEL NON-UNIFORMITY of GLRLM in VOI_edema in Gdzscore 2). |
| 450 | z_score_edema_GLRLMRp_SD | Standard deviation of RUN PERCENTAGE of GLRLM in VOI_edema in Gdzscore 2). |
| 451 | z_score_edema_GLRLMRln_SD | Standard deviation of RUN LENGTH NON-UNIFORMITY in VOI_edema in Gdzscore 2). |
| 452 | z_score_edema_GLRLMLrge_SD | Standard deviation of LOW GRAY LEVEL RUN EMPHASIS in VOI_edema in Gdzscore 2). |
| 453 | z_score_edema_GLRLMHrge_SD | Standard deviation of HIGH GRAY LEVEL RUN EMPHASIS in VOI_edema in Gdzscore 2). |
| 454 | T1_edema_GLCMcorrelation_1 | Correlation of GLCM in VOI_edema in 256-leveled T1-weighted image with offset set to 1. |
| 455 | T1_edema_GLCMcorrelation_2 | Correlation of GLCM in VOI_edema in 256-leveled T1-weighted image with offset set to 2. |
| 456 | T1_edema_GLCMcorrelation_3 | Correlation of GLCM in VOI_edema in 256-leveled T1-weighted image with offset set to 3. |
| 457 | T1_edema_GLCMcorrelation_1_SD | Standard deviation of Correlation of GLCM in VOI_edema in 256-leveled T1-weighted image with offset set to 1. |
| 458 | T1_edema_GLCMcorrelation_2_SD | Standard deviation of Correlation of GLCM in VOI_edema in 256-leveled T1-weighted image with offset set to 2. |
| 459 | T1_edema_GLCMcorrelation_3_SD | Standard deviation of Correlation of GLCM in VOI_edema in 256-leveled T1-weighted image with offset set to 3. |
| 460 | z_score_edema_GLCMcorrelation_1 | Correlation of GLCM in VOI_edema in Gdzscore 2) with offset set to 1. |
| 461 | z_score_edema_GLCMcorrelation_2 | Correlation of GLCM in VOI_edema in Gdzscore 2) with offset set to 2. |
| 462 | z_score_edema_GLCMcorrelation_3 | Correlation of GLCM in VOI_edema in Gdzscore 2) with offset set to 3. |
| 463 | z_score_edema_GLCMcorrelation_1_SD | Standard deviation of Correlation of GLCM in VOI_edema in Gdzscore 2) with offset set to 1. |
| 464 | z_score_edema_GLCMcorrelation_2_SD | Standard deviation of Correlation of GLCM in VOI_edema in Gdzscore 2) with offset set to 2. |
| 465 | z_score_edema_GLCMcorrelation_3_SD | Standard deviation of Correlation of GLCM in VOI_edema in Gdzscore 2) with offset set to 3. |
| 466 | T1_core_GLCMcorrelation_1 | Correlation of GLCM in VOI_core in 256-leveled T1-weighted image with offset set to 1. |
| 467 | T1_core_GLCMcorrelation_2 | Correlation of GLCM in VOI_core in 256-leveled T1-weighted image with offset set to 2. |
| 468 | T1_core_GLCMcorrelation_3 | Correlation of GLCM in VOI_core in 256-leveled T1-weighted image with offset set to 3. |
| 469 | T1_core_GLCMcorrelation_1_SD | Standard deviation of Correlation of GLCM in VOI_core in 256-leveled T1-weighted image with offset set to 1. |
| 470 | T1_core_GLCMcorrelation_2_SD | Standard deviation of Correlation of GLCM in VOI_core in 256-leveled T1-weighted image with offset set to 2. |
| 471 | T1_core_GLCMcorrelation_3_SD | Standard deviation of Correlation of GLCM in VOI_core in 256-leveled T1-weighted image with offset set to 3. |
| 472 | T2_core_GLCMcorrelation_1 | Correlation of GLCM in VOI_core in 256-leveled T2-weighted image with offset set to 1. |
| 473 | T2_core_GLCMcorrelation_2 | Correlation of GLCM in VOI_core in 256-leveled T2-weighted image with offset set to 2. |
| 474 | T2_core_GLCMcorrelation_3 | Correlation of GLCM in VOI_core in 256-leveled T2-weighted image with offset set to 3. |
| 475 | T2_core_GLCMcorrelation_1_SD | Standard deviation of Correlation of GLCM in VOI_core in 256-leveled T2-weighted image with offset set to 1. |
| 476 | T2_core_GLCMcorrelation_2_SD | Standard deviation of Correlation of GLCM in VOI_core in 256-leveled T2-weighted image with offset set to 2. |
| 477 | T2_core_GLCMcorrelation_3_SD | Standard deviation of Correlation of GLCM in VOI_core in 256-leveled T2-weighted image with offset set to 3. |
| 478 | T1Gd_core_GLCMcorrelation_1 | Correlation of GLCM in VOI_core in 256-leveled Gadolinium enhanced T1-weighted image with offset set to 1. |
| 479 | T1Gd_core_GLCMcorrelation_2 | Correlation of GLCM in VOI_core in 256-leveled Gadolinium enhanced T1-weighted image with offset set to 2. |
| 480 | T1Gd_core_GLCMcorrelation_3 | Correlation of GLCM in VOI_core in 256-leveled Gadolinium enhanced T1-weighted image with offset set to 3. |
| 481 | T1Gd_core_GLCMcorrelation_1_SD | Standard deviation of Correlation of GLCM in VOI_core in 256-leveled Gadolinium enhanced Gadolinium enhanced T1-weighted image with offset set to 1. |
| 482 | T1Gd_core_GLCMcorrelation_2_SD | Standard deviation of Correlation of GLCM in VOI_core in 256-leveled T2-weighted image with offset set to 2. |
| 483 | T1Gd_core_GLCMcorrelation_3_SD | Standard deviation of Correlation of GLCM in VOI_core in 256-leveled T2-weighted image with offset set to 3. |
| 484 | z_score_core_GLCMcorrelation_1 | Correlation of GLCM in VOI_core in Gdzscore 2) with offset set to 1. |
| 485 | z_score_core_GLCMcorrelation_2 | Correlation of GLCM in VOI_core in Gdzscore 2) with offset set to 2. |
| 486 | z_score_core_GLCMcorrelation_3 | Correlation of GLCM in VOI_core in Gdzscore 2) with offset set to 3. |
| 487 | z_score_core_GLCMcorrelation_1_SD | Standard deviation of Correlation of GLCM in VOI_core in Gdzscore 2) with offset set to 1. |
| 488 | z_score_core_GLCMcorrelation_2_SD | Standard deviation of Correlation of GLCM in VOI_core in Gdzscore 2) with offset set to 2. |
| 489 | z_score_core_GLCMcorrelation_3_SD | Standard deviation of Correlation of GLCM in VOI_core in Gdzscore 2) with offset set to 3. |

1. Prewitt filtering was performed by applying first order horizontal Gx and vertical Gy differentiation and by calculating the magnitude G, where Gx and Gy stands for the horizontal and vertical gradient of the image respectively and A for the original two-dimensional gray scale image

, ,

1. Gdzscore image was created by visualizing the magnitude of enhancement calculated from both 256-leveled non-enhanced and Gadolinium-enhanced T1-weighted images. 256-leveled Gadolinium-enhanced T1-weighted images was plotted as a function of 256-leveled non-enhanced T1-weighted images in whole brain. Linear regression fitting was applied to the data obtained, which can be expressed as follows;

where (*GdT1WI*) and (*T1WI*) are 256-leveled Gadolinium-enhanced and non-enhanced T1-weighted images. By solving *α* and *β*, one can now determine the linear correlation of 256-leveled Gadolinium-enhanced and non-enhanced T1-weighted images.

Next, the magnitude of deviation from the above solved linear regression line for any particular voxel (*i*) can be expressed as follows:

where (*GdT1WI*)*i* and (*T1WI*) *i* are the 256-leved values of voxel (*i*) in Gadolinium-enhanced and non-enhanced T1-weighted images.

Finally, the Gdzscore of each data point was defined as follows

where *μ* and *σ* are the means and standard deviation of *deviationi* in the whole brain
