# Supplementary figures and images for: Radiomics-based preoperative survival prediction in newly diagnosed glioblastoma: A multicohort study with internal and external validation
Source: Neurooncol Adv. 2026 Mar 13;8(1):vdag068. doi: 10.1093/noajnl/vdag068 (PMC13063838; doi:10.1093/noajnl/vdag068)

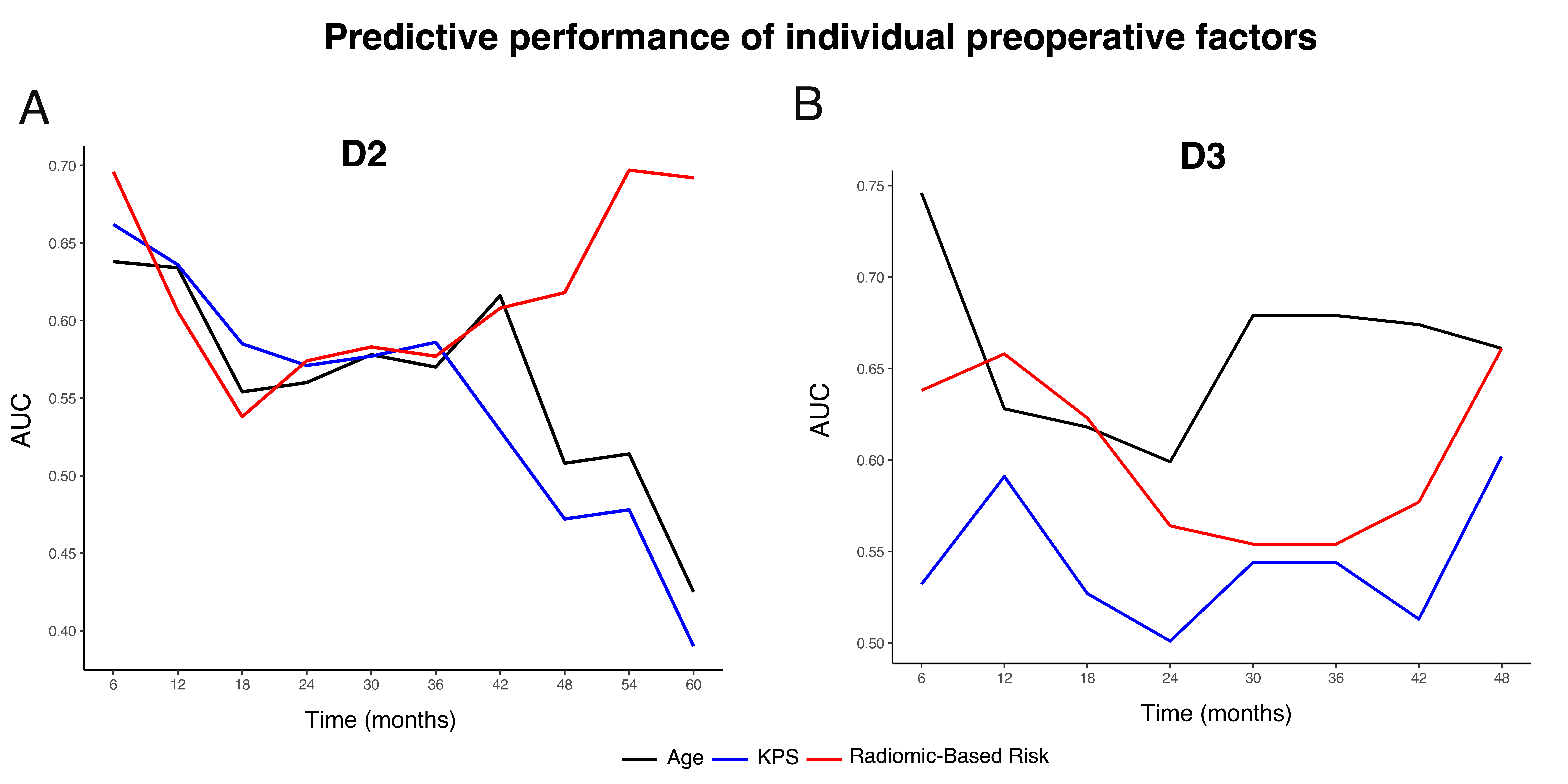

Supplement: vdag068_Supplementary_Data [file vdag068_supplementary_data.zip › Supplementary_Data/Supplementary Figure S2.tiff]
